# BDNF/trkB Induction of Calcium Transients through Ca_v_2.2 Calcium Channels in Motoneurons Corresponds to F-actin Assembly and Growth Cone Formation on β2-Chain Laminin (221)

**DOI:** 10.3389/fnmol.2017.00346

**Published:** 2017-10-30

**Authors:** Benjamin Dombert, Stefanie Balk, Patrick Lüningschrör, Mehri Moradi, Rajeeve Sivadasan, Lena Saal-Bauernschubert, Sibylle Jablonka

**Affiliations:** Institute of Clinical Neurobiology, University of Wuerzburg, Wuerzburg, Germany

**Keywords:** BDNF, trkB, Ca_v_2.2, F-actin, motor axon, growth cone

## Abstract

Spontaneous Ca^2+^ transients and actin dynamics in primary motoneurons correspond to cellular differentiation such as axon elongation and growth cone formation. Brain-derived neurotrophic factor (BDNF) and its receptor trkB support both motoneuron survival and synaptic differentiation. However, in motoneurons effects of BDNF/trkB signaling on spontaneous Ca^2+^ influx and actin dynamics at axonal growth cones are not fully unraveled. In our study we addressed the question how neurotrophic factor signaling corresponds to cell autonomous excitability and growth cone formation. Primary motoneurons from mouse embryos were cultured on the synapse specific, β2-chain containing laminin isoform (221) regulating axon elongation through spontaneous Ca^2+^ transients that are in turn induced by enhanced clustering of N-type specific voltage-gated Ca^2+^ channels (Ca_v_2.2) in axonal growth cones. TrkB-deficient (*trkBTK*^−/−^*)* mouse motoneurons which express no full-length trkB receptor and wildtype motoneurons cultured without BDNF exhibited reduced spontaneous Ca^2+^ transients that corresponded to altered axon elongation and defects in growth cone morphology which was accompanied by changes in the local actin cytoskeleton. *Vice versa*, the acute application of BDNF resulted in the induction of spontaneous Ca^2+^ transients and Ca_v_2.2 clustering in motor growth cones, as well as the activation of trkB downstream signaling cascades which promoted the stabilization of β-actin via the LIM kinase pathway and phosphorylation of profilin at Tyr129. Finally, we identified a mutual regulation of neuronal excitability and actin dynamics in axonal growth cones of embryonic motoneurons cultured on laminin-221/211. Impaired excitability resulted in dysregulated axon extension and local actin cytoskeleton, whereas upon β-actin knockdown Ca_v_2.2 clustering was affected. We conclude from our data that in embryonic motoneurons BDNF/trkB signaling contributes to axon elongation and growth cone formation through changes in the local actin cytoskeleton accompanied by increased Ca_v_2.2 clustering and local calcium transients. These findings may help to explore cellular mechanisms which might be dysregulated during maturation of embryonic motoneurons leading to motoneuron disease.

## Introduction

It is widely recognized that neurotrophic factor signaling contributes to motoneuron survival (Arakawa et al., 1990; Sendtner et al., 1991, 1992; Hughes et al., 1993; Henderson et al., 1994; Pennica et al., 1995, 1996). Motoneuron differentiation strongly depends on neuronal excitability characterized by spontaneous Ca^2+^ transients which constitute an evolutionary conserved phenomenon (O'Donovan and Landmesser, 1987; Gu and Spitzer, 1995; Spitzer et al., 2000; Hanson and Landmesser, 2004; Spitzer, 2006; Jablonka et al., 2007; Wang et al., 2009). These spontaneous Ca^2+^ elevations at growth cones are established through the precise localization and accumulation of voltage-gated calcium channels (VGCCs), as well as the regulatory influence of matrix proteins like laminins (Nishimune et al., 2004; Jablonka et al., 2007). Laminins consist of three chains, i.e., α, β and γ (Patton et al., 1997; Aumailley et al., 2005). There are five α-chain and three γ-chain isoforms (Aumailley et al., 2005). The β-chain is expressed as β1, β2, and β3 (Aumailley et al., 2005). In the synaptic cleft isoforms comprising the β2-chain predominate. Mice deficient of β2-chain laminins display tremendous morphological abnormalities in neuromuscular endplates (Noakes et al., 1995). Upon depletion of β1- or γ1-chains from laminin-111 or -211, the basement membrane fails to form (Colognato and Yurchenco, 2000) and causes a severe peripheral neuropathy characterized by amyelination and reduced proliferation (Chen and Strickland, 2003; Yang et al., 2005; Yu et al., 2005). Thus, β1-chain laminins are termed “Schwann cell-specific”, whereas β2-chain laminins are specified as “synapse-specific.” Beta 2-chain laminins are reported to associate with the pore-forming (Ca_v_) subunit of N- and P/Q type calcium channels (Nishimune et al., 2004). In embryonic motoneurons this interaction leads to Ca_v_2.2 accumulation which corresponds to increased spontaneous Ca^2+^ influx at axonal growth cones modulating axonal extension which is not observed on β1-chain laminins such as laminin-111 (Jablonka et al., 2007). In turn, axon growth also depends on an organized cytoskeleton at axonal growth cones with actin filaments playing a central role in this scenario. Several studies have demonstrated that in forebrain neurons neurotrophins such as BDNF or NGF increase β-actin mRNA transport from the cell body to the periphery (Zhang et al., 1999, 2001; Willis et al., 2007). Furthermore, local translation is induced through Src-dependent phosphorylation of the β-actin mRNA zipcode binding protein 1 (ZBP1) leading to growth cone turning in correspondence to cellular Ca^2+^ availability (Zhang et al., 1999, 2001; Hüttelmaier et al., 2005; Yao et al., 2006; Sasaki et al., 2010).

In motoneurons it is unclear whether these effects are specific for BDNF or if other growth factors such as CNTF and GDNF are equally important. It has been reported that the application of BDNF, CNTF and/or GDNF to isolated primary motoneurons from chick, human, rat and *Xenopus* promotes survival (Sendtner et al., 1990, 1992; Henderson et al., 1994; Li et al., 1994), up-regulated the cholinergic phenotype (Wong et al., 1993), stimulates Choline Acetyltransferase (ChAT) activity (Kato and Lindsay, 1994) and leads to increased acetylcholine release in quantal packets (Liou et al., 1997). BDNF, as a member of the neurotrophin family, acts through the tropomyosin-related kinase (trkB) family of receptor tyrosine kinases (Klein et al., 1989, 1991; Middlemas et al., 1991; Soppet et al., 1991). GDNF is a founding member of the GDNF family of ligands (GFL) which binds to GDNFRα1 and 2 mediating the activation of c-ret (rearranged during transfection) tyrosine kinase (Jing et al., 1996; Treanor et al., 1996). CNTF belongs to the IL-6 family and binds to CNTFRα which is closely related to the IL-6 receptor (Davis et al., 1991). Signal transduction is conveyed by gp130 and LIFRβ which together with CNTFRα form the so-called tripartite complex (Taga et al., 1989; Hibi et al., 1990; Gearing et al., 1991; Davis et al., 1993; Stahl and Yancopoulos, 1994).

The impact of each of these neurotrophic factors on cellular differentiation such as axon elongation and growth cone formation has not been resolved completely in embryonic motoneurons. It also remains to be investigated to which degree extracellular stimuli like β2-chain laminins complement neurotrophic factor signaling in order to induce the formation of axons and growth cones through changes in local neuronal excitability and actin dynamics. In this study we have conducted a series of morphological and functional comparative analyses of p75^NTR^-enriched embryonic *trkBTK*^−/−^ and BDNF-deprived motoneurons cultured on a laminin isoform carrying the β2-chain, i.e., laminin-221/211, including control experiments on a β1-chain laminin isoform, i.e., laminin-111. Our data provide evidence that most notably BDNF/trkB signaling appears necessary and sufficient to actuate and sustain motor axon elongation and growth cone formation through Ca_v_2.2 accumulation, spontaneous Ca^2+^ influx and modulation of actin dynamics at axonal growth cones in collaboration with the β2-chain of laminin 221.

## Material and methods

### Animals

CD-1, C57Bl/6 and *trkBTK*^+/−^ (Klein et al., 1993) mice were housed in the central animal breeding facility of the Institute of Clinical Neurobiology at the University Hospital Wuerzburg ensuring controlled conditions, i.e., food and water in abundant supply, 20–22°C, 55–65% humidity and 12:12 h light/dark cycle. All experiments were carried out strictly corresponding to the German federal regulations on animal protection and the rules of the Association for Assessment and Accreditation of Laboratory Animal Care, with the explicit approval of the local veterinary authority (Veterinaeramt der Stadt Wuerzburg) and the Committee on the Ethics of Animal Experiments (Regierung von Unterfranken), Wuerzburg. Genotyping of *trkBTK*^+/+^, *trkBTK*^+/−^ and *trkBTK*^−/−^ mouse embryos was conducted using the following primers: trkB sense—5′ TCG CGT AAA GAC GGA ACA TGA TCC 3′; trkB wild type—5′ AGA CCA TGA TGA GTG GGT CGC C 3′; trkB knockout—5′ GAT GTG GAA TGT GTG CGA GGC C 3′.

### Primary motoneuron culture

The ventrolateral part of the lumbar spinal cord of E13 mouse embryos was isolated, and spinal motoneurons were enriched via p75^NTR^ panning (Wiese et al., 1999, 2010). Glass coverslips or cell culture dishes were coated with poly-ornithine (Sigma-Aldrich) and laminin-221/211 (Chemicon, Millipore) or laminin-111 (Sigma-Aldrich), respectively. Laminin-221/211 is composed of laminin-221 and -211 due to the filtration procedure of the manufacturer (see Chemicon, Millipore). Laminin-221 carries a α2-, β2-, and γ1-chain, whereas laminin-211 consists of a α2-, β1-, and γ1-chain. Laminin-111 comprises a α1-, β1-, and γ1-chain (Aumailley et al., 2005). Motoneurons were cultured for 5 or 7 days *in vitro* at 37°C in a 5% atmosphere. After 5 days *in vitro* (DIV5) growth cones were analyzed, whereas after 7 days (DIV7) neurite outgrowth and soma size were determined. Motoneuron medium comprised Neurobasal Medium (Gibco), 2% horse serum (heat-inactivated, Linearis), 500 μM GlutaMAX™-I (Gibco) and 2% B-27 (Gibco). Medium was changed at day 1 *in vitro* and then every second day. To ensure cell survival *trkBTK*^+/+^ and *trkBTK*^−/−^ motoneurons were kept in culture with 10 ng/ml BDNF and CNTF each (Wiese et al., 2007). For comparative analyses of neurotrophic factor potency wild type motoneurons were incubated with 1 ng/ml BDNF, CNTF or GDNF. To examine the subcellular distribution of trkB and c-ret 1 ng/ml BDNF, CNTF and GDNF were added to the medium. For BDNF pulse experiments cells were cultured with 1 ng/ml BDNF and CNTF for 4 days *in vitro*. Motoneurons were washed twice with neurobasal medium. Then, motoneuron medium with 1 ng/ml CNTF was applied for further 16 h before acute application of 10 ng/ml (ICC/IF analyses) or 20 ng/ml (biochemical analyses) BDNF. Subsequently, cells were fixed with 4% paraformaldehyde (PFA) for 15 min at room temperature (RT) or protein lysates for Western blot analyses were produced in accordance to the applied experimental design. Optionally, motoneurons were pre-incubated with pharmacological inhibitors, i.e., 0.1 μM Src kinase inhibitor cocktail 1 (PP1, Calbiochem) or 0.5 μM cytochalasin D (CytD, Sigma-Aldrich), for 30 min before BDNF was added. Since PP1 was dissolved in 1% DMSO, we tested if this toxic substance *per se* might interfere with the observed effects of BDNF with respect to Ca_v_2.2 clustering which was not the case. Transduction with lentiviruses was performed directly before plating using previously published pSIH-H1 constructs for β-actin knockdown and re-expression (Moradi et al., 2017). In brief, p75-enriched and concentrated motoneurons were incubated with shRNA oligonucleotides targeting the 3′ UTR of β-actin (shActβ) for 8 min at RT. Successful transduction was monitored by GFP expression. The luciferase pSIH-H1 vector (shLUC) served as reference control. The Actβ rescue construct “Rescue” comprised the coding sequence of mouse Actβ and a shRNA-resistant version of the corresponding 3′ UTR which had been cloned into an expression vector under the ubiquitin promotor. This ubiquitin-Actβ-rescue cassette was excised and re-cloned into the pSIH-H1 construct containing shActβ.

### Survival analysis of embryonic motoneurons

The survival rate of isolated embryonic mouse motoneurons was assessed by determining the cell number of three randomly chosen fields for each analyzed coverslip at day 1 and 7 *in vitro*. The average number of cells per field at day 1 *in vitro* was set as 100%.

### Immunofluorescent stainings of embryonic motoneurons

Immunocytochemical and -fluorescent (ICC/IF) stainings of cultured motoneurons were carried out as reported previously with minor modifications (Dombert et al., 2014). Depending on the experimental design, motoneurons were treated with ice-cold acetone for 2 min or with 0.3% TritonX-100 for 20 min to permeabilize cells. For detection of trkB and Ca_v_2.2 0.1% Tween-20 was used during the staining procedure. The following primary and secondary antibodies were applied in this study: Ca_v_2.2 (1:500, N-terminal, rabbit from Sigma-Aldrich (#C1478), guinea pig from Synaptic Systems (#152305)), trkB (1:1000, Millipore, #07-225), tau (1:750, Sigma-Aldrich, #T6402), APP (1:300, Acris Antibodies, #AM09000PU-N), Glu-α-tubulin (1:1000, Synaptic Systems, #302011), Tyr-YL1/2-tubulin (1:2000, Abcam, #ab6160), synaptophysin (1:1000, Synaptic Systems, #101004), β-actin (1:500, GeneTex AC-15, #GTX26276), cofilin (1:300, Abcam, #ab54532), phospho-cofilin (Ser3) (1:600, Abcam, #ab47281), phospho-profilin (Tyr129) (1:300, ECM Biosciences, #PK6930), c-ret (1:200, Neuromics, #GT15002) and phospho-trk (1:500, Y705, Abcam, #ab52191), donkey anti-goat-Cy2 (1:200, Jackson Immunoresearch (JI)), donkey anti-rabbit-Cy2 (1:600, JI), donkey anti-rabbit-Cy3 (1:700, JI), donkey anti-rabbit-DyLight649 (1:600, JI), goat anti-rabbit-Cy5 (1:500, Invitrogen), donkey anti-mouse-Alexa488 (1:500, Invitrogen), donkey anti-mouse-Cy5 (1:600, JI), goat anti-mouse-Cy5 (1:500, JI), donkey anti-guinea pig-Cy2 (1:500, JI), donkey anti-guinea pig-Cy3 (1:500, JI), donkey anti-guinea pig-Cy5 (1:500, JI), donkey anti-rat-Alexa488 (1:600, Molecular probes), donkey anti-rat-Cy3 (1:600, JI), goat anti-rat-DyLight649 (1:400, JI), phalloidin-Alexa546 (1:40, ThermoFisher Scientific, #A22283) and phalloidin-Acti stain 670 (1:50, Cytoskeleton, #PHDN1-A). Coverslips were embedded in mowiol (Sigma-Aldrich) and imaged with a confocal microscope. Specificity of the anti-trkB antibody purchased from Millipore was validated in cultured motoneurons by trkB knockdown via lentiviral gene transfer (Figure S5B) using a previously published target sequence (5′-TTGTGGATTCCGGCTTAAA-3′) (Bartkowska et al., 2007; Puehringer et al., 2013).

### FISH of cultured primary motoneurons

Fluorescence *in situ* hybridization (FISH) was conducted according to the manufacturer's instructions (GeneDetect, PerkinElmer) and previous reports (Rossoll et al., 2003; Jablonka et al., 2007). Anti-sense (5′-GCCGATCCACACGGAGTACTTGCGCTCAGGAGGAGCAAT GATCTTGAT-3′) and sense (5′-CGGCTAGGTGTGCCTCATGAACGCGAGTCCTCCT CGTTACTAGAACTA-3′) 3′-biotinylated oligonucleotides against actin mRNA (100 ng/ml) were added for 16 h at 37°C. Visual detection was carried out by using the tyramide signal amplification (TSA) system (PerkinElmer) and TRITC-streptavidin (Zymed, 1:100) for fluorescent labeling.

### Calcium imaging

For Ca^2+^ imaging approaches the calcium indicator Oregon Green 488 BAPTA-1 AM (OGB, Invitrogen 06807) was mixed with 8.9 ml 20% Pluronic F-27/DMSO in an ultrasound sonifier (Bandelin) for 2 min. Motoneurons were incubated with the calcium indicator for 13 min at 37°C and 5% CO_2_ which had been diluted 1: 1000 in a calcium imaging buffer comprising 135 mM NaCl, 1 mM MgCl_2_, 10 mM HEPES, 1 mM CaCl_2_, 6 mM KCl, and 5.5 mM glucose. Afterwards, the coverslips containing the motoneurons were transferred to a heated chamber filled with 2 ml calcium imaging buffer. Recordings were produced with the help of a confocal microscope (BX51 WI, Olympus), a 40 × objective (LUMPlanFI/IR, 0.8 W, Olympus) and a camera (Rolera-XR, Qimaging) which generated time-lapse images with a frequency of 2.5 Hz and an exposure time of 400 ms. Images were displayed in real time by the StreamPix (Norpix) software. Motoneurons were irradiated continuously with a LED light source (Visitron Systems) using the following filter settings: excitation (482 ± 35 nm), dichroitic filter (506 nm) and emission filter (536 ± 40 nm). Analyses were carried out with the ImageJ plugins “Intensity vs. Time Monitor” and “Time Series Analyzer V2.0” which allowed the determination of activity events by measuring calcium influx in defined regions of interest, i.e., growth cones. Growth cones with at least one spike per time period were specified as “active growth cones.” A spike is defined as an event which exceeds at least twofold the background noise and shows no deflection to the negative. In the context of transient pulse experiments the same axonal growth cones of embryonic motoneurons were analyzed before and directly after BDNF application (10 ng/ml). Treatment with 30 nM ω-conotoxin (CTX, Sigma-Aldrich) significantly reduced the number of measured activity events in growth cones of three independent experiments validating the identity of these spontaneous calcium transients (Figure S1A).

### Image acquisition, processing and analysis

In this study, the Leica confocal systems SP2 and SP5 were used for image acquisition. Fluorescence intensity measurement was performed by applying identical photomultiplier, laser intensity, magnification, offset and objective settings. Signal intensities were measured from raw images as mean gray values per pixel based on quantum levels (QL) per pixel using the Leica LAS AF LITE Software (Leica). Values were presented as arbitrary units per area or pixel, respectively. Background intensity was determined and subtracted for each image. Growth cones with a pronounced peripheral region, e.g., protrusions, were considered for morphological and statistical analysis. Intracellular distribution of trkB and c-ret within growth cones was assessed by calculating the ratio of peripheral versus central growth cone regions considering the sizes of the corresponding regions of interest. Final processing of all images was carried out with ImageJ (MacBiophotonics) and Illustrator CS5 (Adobe). For qualitative presentation brightness and contrast were equally enhanced for consistent images without affecting image information.

### Data analyses and statistics

If not indicated otherwise, at least three independent experiments were conducted for each statistical analysis and data are depicted as scatter plots showing the mean ± standard error of the mean (SEM). The number of statistically assessed biological and technical replicates is represented by “n.” In turn, each statistical number derives from the median of one analyzed group, e.g., 16 measured growth cones per technical/biological replicate. Thus, “N” indicates the total number of scored specimens, e.g., motoneuron somata, growth cones, axons or actin movements. For statistical analysis either “n” or “N” was considered as described in the text and the provided statistics-file. Two sets of data were compared with each other by unpaired *t*-test or Mann-Whitney U-test, the latter if values were not distributed normally. If more than two groups were analyzed, one-way ANOVA followed by Tukey's after testing or Kruskal-Wallis analysis with post-hoc Dunn's multiple comparison tests was applied, the latter again when values were not displaying a Gaussian distribution. Statistical analyses were conducted using GraphPad Prism 6. Statistical significance was defined as *p* < 0.05. Each p value is depicted in the corresponding graph. Data from different experiments were pooled when values of consistent control groups were comparable to each other. Signal intensities from control specimens were normalized to “1.” Densitometric intensities in western blots were normalized against the corresponding control proteins. Exclusively motoneurons with one axon were considered for analysis. Only the longest axon branch was measured for axon length, axon collaterals were not considered for the analysis. Motoneurons were only scored when designated axons were at least three times longer than the corresponding dendrites ensuring an unambiguous distinction between axons and dendrites. A list with detailed data about the obtained statistics is provided as a separate pdf-file within the Supplementary Material.

### Western blot analysis

Primary motoneurons were washed once with PBS, lysed with 2 x Laemmli buffer (125 mM Tris, pH 6.8, 4% SDS, 10% β-mercaptoethanol, 20% glycerol and 0.004% bromophenol blue), boiled for 5 min at 99°C and sonified. Lysates were subjected to SDS-PAGE, blotted onto nitrocellulose membrane and blocked with 5% milk powder in TBST for 1 h at room temperature. Subsequently, blots were incubated with primary antibodies overnight. The next day, blots were incubated with appropriate horseradish-peroxidase conjugated secondary antibodies for 1 h at room temperature and signals were detected on X-ray film (Fuji super RX) using enhanced chemiluminescence. X-ray films were scanned and quantified by densitometry analysis with ImageJ (National Institutes of Health). The following primary antibodies were used: anti-LIM kinase (Acris #TA503012), anti-phospho LIM kinase (Cell Signaling #3841), anti-phospho-cofilin (Abcam #AB12866), anti-phospho-AKT (Cell Signaling, #9275), anti-AKT (Cell Signaling #9272), anti-MAPK (Cell Signaling #4696, clone L34F12), anti-phospho-MAPK (Cell Signaling #E10, clone E10), pTrk (Y704/705 for pTrkB) (Cell Signaling #4621), trkB (Millipore #07-225), Calnexin (Enzo, ADI-SPA-860), β-actin (GeneTex #GTX26276, clone AC-15).

### G- to F-actin separation and western blotting

G- to F-actin separation was conducted as described recently (Sivadasan et al., 2016; Moradi et al., 2017). Non-pulsed and BDNF-pulsed primary motoneurons were washed two times with warm PBS prior to cell lysis using actin stabilization buffer comprising 0.1 M Pipes, pH 6.9, 30% glycerol (vol/vol), 5% DMSO (vol/vol), 1 mM MgSO_4_, 1 mM EGTA, 1% Triton X-100 (vol/vol), 1 mM ATP, complete protease inhibitor and phosphatase inhibitor. Lysates were kept at 37°C for 10 min. Cell extracts were ultracentrifuged at 100 000 g for 75 min at 37°C. The supernatant containing G-actin was recovered, whereas the pellet comprising F-actin was resuspended with RIPA buffer (50 mM Tris pH 8.0, 1% NP40, 0.5% sodium deoxycholate, 0.1% SDS, 150 mM NaCl, 2 mM EDTA and 50 mM NaF). Equal amounts of proteins as determined by BCA protein assay kit (Pierce BCA Protein Assay Kit) were applied for subsequent western blot analysis using the following primary and secondary antibodies: mouse monoclonal anti-Actβ (1:4,000, GeneTex), goat polyclonal anti-calnexin (1:5,000, Acris), donkey anti-mouse IgG (1:10,000, Jackson ImmunoResearch), donkey anti-goat IgG (1:10,000, Jackson ImmunoResearch).

### Live cell imaging

For measuring actin dynamics a GFP-tagged cytoplasmic actin construct which incorporates into actin-containing structures was used as reported previously (Fischer et al., 1998; Sivadasan et al., 2016). Transduced motoneurons were cultured on laminin-221/211 coated 35-mm μ-dishes (Ibidi) for 4 days *in vitro* with 1 ng/ml BDNF and CNTF at a density of 15,000 cells. BDNF was removed by washing the cells three times with warm phenol red-free Neurobasal medium and motoneurons were cultured for further 16 h with phenol red-free medium comprising 1 ng/ml CNTF. At day 5 live cell imaging of the same growth cones was performed before and after BDNF pulse (10 ng/ml) using the Eclipse TE2000 microscope (Nikon) equipped with a 60 x Plan Apochromat NA 1.4 immersion objective and heated stage chamber (TOKAI HIT CO., LTD) at 37°C and 5% CO_2_. GFP was excited by a 470 nm cool LED PE-100 light source at 2–4% light emission intensity. Emission was filtered through a 493/574 nm dual-band beam splitter and recorded using an Orca Flash 4.0 V2 camera (12-bit, 1,024 × 1,024, Hamamatsu Photonics). Actin dynamics of axonal growth cones originating from the corresponding central points were assessed by time-lapse imaging for 20 min at 15 s intervals. To determine the central point of a growth cone a maximum projection of all 81 frames was produced and the central point was localized halfway from axon shaft to distal protrusions. The selected central point was then restored to the time-lapse movie and multiple kymographs were produced and analyzed from all 81 frames applying the ImageJ Kymograph plug-in. The x axis provides information about time (in seconds) and the y axis about distance (in μm). Thus, the velocity of actin movement in axon terminals was calculated as delta length in μm divided by the time in seconds for each pick (15 s) through the length measurement of each pick from the x and y axes. After imaging the BDNF-pulsed axonal growth cones, motoneurons were again washed three times with phenol red-free neurobasal medium and incubated for further 24 h with phenol red-free motoneuron medium comprising 1 ng/ml CNTF. At day 6 the same procedure was repeated and the results were comparable and reproducible. Statistical analysis was carried out with actin movement data pooled from day 5 and day 6 old motoneurons.

## Results

### Defective Ca_v_2.2 clustering and decreased frequency of local spontaneous Ca^2+^ transients in axonal growth cones of *trkBTK^−/−^* motoneurons cultured on laminin-221

**Figure 1:**
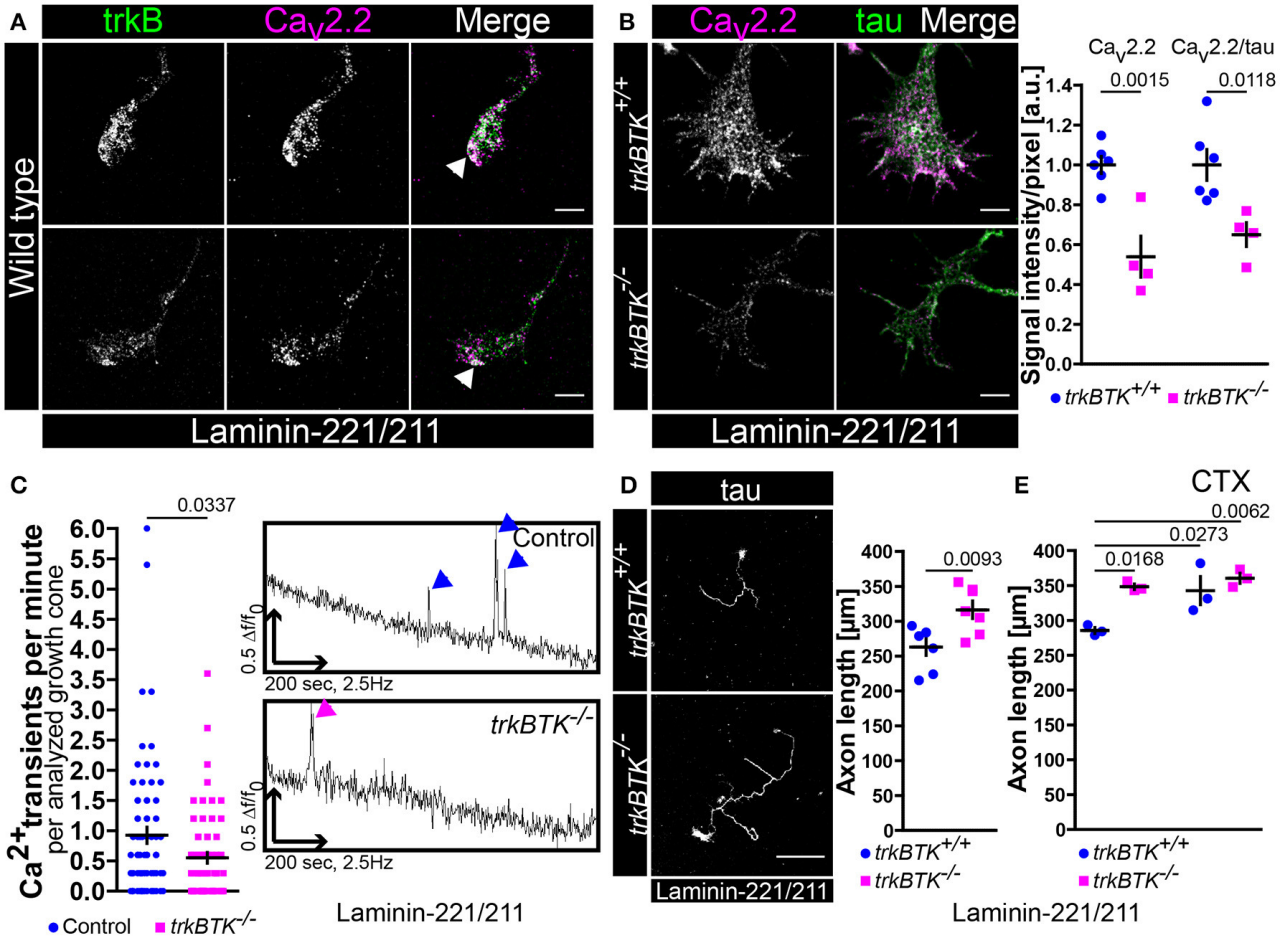
Decrease of Ca_v_2.2 accumulations and spontaneous Ca^2+^ transients in growth cones of *trkBTK*^−/−^ motoneurons corresponds to altered axon growth on laminin-221. **(A)** Representative images of axonal growth cones of wild type motoneurons cultured on laminin-221/211 for 5 days *in vitro* in the presence of BDNF and CNTF. TrkB receptors (green) and Ca_v_2.2 calcium channels (magenta) were closely localized in growth cone protrusions, highlighted by white arrowheads (scale bar: 5 μm). **(B)** In *trkBTK*^−/−^ growth cones Ca_v_2.2 accumulation (magenta) was affected in growth cone tips, whereas tau levels (green) were not altered (scale bar: 5 μm). Statistical analysis of Ca_v_2.2 immunoreactivity in *trkBTK*^−/−^ axonal growth cones (0.54 ± 0.1, Q_2_ 0.47, *n* = 4, *N* = 66) in comparison to wild type controls (1.00 ± 0.04, Q_2_ 1.01, *n* = 6, *N* = 78) revealed a significant difference (*p* = 0.0015). Similar findings were obtained by normalizing Ca_v_2.2 against the internal reference protein tau (*trkBTK*^+/+^ 1.00 ± 0.08, Q_2_ 0.95; *trkBTK*^−/−^ 0.65 ± 0.06, Q_2_ 0.67; *p* = 0.0118) **(C)** The structural phenotype was accompanied by significant differences in the frequency of spontaneous calcium transients between control and *trkBTK*^−/−^ growth cones (Control 0.92 ± 0.13, Q_2_ 0.60, *N* = 72; *trkBTK*^−/−^ 0.55 ± 0.09, Q_2_ 0.30, *N* = 64; *p* = 0.0337). Representative recordings of control (upper trace, blue arrowheads) and *trkBTK*^−/−^ (lower trace, magenta arrowheads) growth cones showed a reduced number of calcium spikes when trkB signaling is impaired. **(D)** Wild type and *trkBTK*^−/−^ motoneurons were cultured on laminin-221/211 for 7 days *in vitro* in the presence of BDNF and CNTF and stained against tau (scale bar: 150 μm). TrkB mutant cells (316.4 ± 12.65 μm, Q_2_ 314.5 μm, *n* = 7, *N* = 298) grew longer axons than controls (262.9 ± 11.79 μm, Q_2_ 278.8 μm, *n* = 7, *N* = 283, *p* = 0.0093) **(E)** Treatment with 30 nM ω-conotoxin (CTX) caused enhanced axonal elongation in wild type motoneurons, but not in *trkBTK*^−/−^ cells.

In order to investigate whether impaired BDNF/trkB signaling affects axon elongation and growth cone formation we started to culture embryonic *trkBTK*^−/−^ motoneurons on synapse specific laminin-221/211 for 5 days (Figure 1). In axonal growth cones of wild type motoneurons trkB receptors and Ca_v_2.2 calcium channels clustered in close proximity particularly at axon tips (Figure 1A, Figure S1B). *TrkBTK*^−/−^ motoneurons developed growth cones with deficits in Ca_v_2.2 accumulation at the corresponding protrusions as determined by significantly reduced Ca_v_2.2 immunoreactivity (Figure 1B). Tau protein levels were not altered (Figure 1B). Furthermore, there seemed to be no difference in Ca_v_2.2 signal intensity between control and *trkBTK*^−/−^ motoneurons when cultured on laminin-111 (Figure S1C). In fact, an enrichment of Ca_v_2.2 immunofluorescence in axonal growth cones of control motoneurons cultured on laminin-221/211 was observed in comparison to laminin-111 (Figure S1C). This alteration was not detectable in *trkBTK*^−/−^ growth cones (Figure S1C). The structural phenotype on laminin-221/211 was accompanied by functional abnormalities in local calcium signaling (Figure 1C). Control and trkB mutant motoneurons cultured for 5 days on laminin-221/211 were loaded with Oregon Green dyes and subjected to calcium imaging over a time period of 200 s. Spontaneous Ca^2+^ transients at the growth cone were identified and assessed as prominent spikes (Figure 1C, right panel). In growth cones of *trkBTK*^−/−^ motoneurons the frequency of spontaneous calcium transients was significantly reduced (Figure 1C, left panel) emphasizing structural and functional defects in local calcium signaling when trkB signaling appears impaired.

We know from previous studies that axon extension is mediated by direct association of Ca_v_ subunits with laminin β2-chains when motoneurons are cultured on synapse-specific laminin-221 (Nishimune et al., 2004; Jablonka et al., 2007). This Ca_v_2.2-β2-chain interaction results in Ca_v_2.2 accumulation and spontaneous Ca^2+^ influx which reflects a differentiation signal in the growth cone suppressing axonal outgrowth (Porter et al., 1995; Jablonka et al., 2007). Since Ca_v_2.2 clustering and local calcium signaling appeared impaired when trkB/BDNF signaling was compromised we investigated in a next step whether axon growth was altered in *trkBTK*^−/−^ motoneurons after culturing them on laminin-221/211 for 7 days (Figure 1D). In comparison to wild type controls, *trkBTK*^−/−^ motoneurons grew longer axons indicating impaired recognition of the aforementioned “growth arrest” signal provided by the association of laminin β2-chains and Ca_v_2.2 calcium channels at axonal growth cones (Figure 1D). Soma size, number of primary dendrites, mean dendrite length and total dendrite length was not affected emphasizing a mainly axonal phenotype (Figure S2A). Treatment with the specific Ca_v_2.2 channel blocker ω-conotoxin (30 nM) until DIV7 (Jablonka et al., 2007) resulted in enhanced axonal elongation in wild type, but not in *trkBTK*^−/−^ motoneurons (Figure 1E). Furthermore, this concentration of CTX during a culture period of 5 days was able to significantly reduce the measured spontaneous Ca^2+^ transients in growth cones of motoneurons cultured on laminin-221/211 (Figure S1A).

### BDNF enhances Ca_v_2.2 clustering and local calcium transients in growth cones of embryonic motoneurons on laminin-221

**Figure 2:**
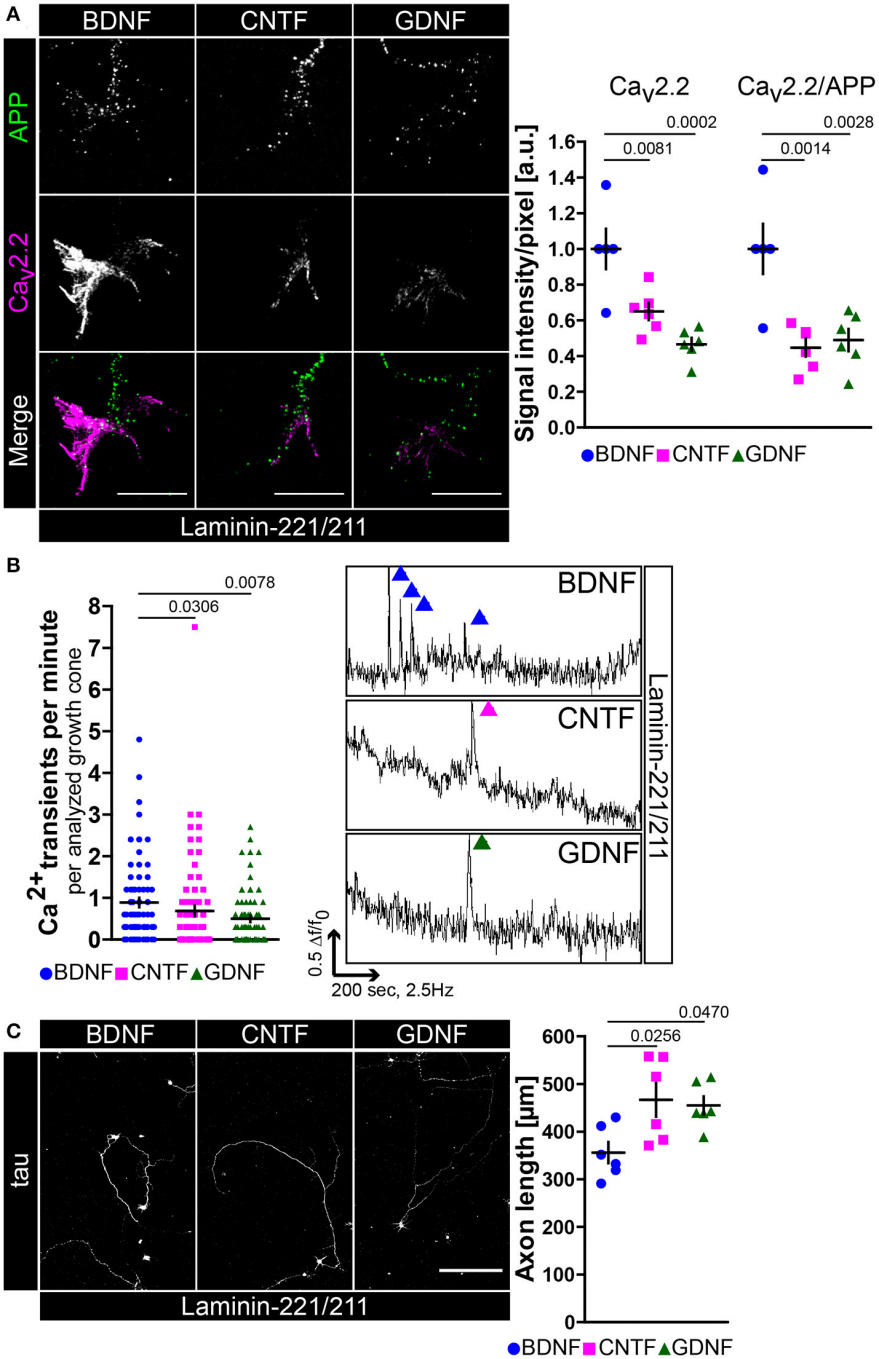
BDNF is necessary for Ca_v_2.2 accumulation and local Ca^2+^ transients in growth cones of motor axons on laminin-221. **(A)** Images of axonal growth cones of wild type motoneurons cultured on laminin-221/211 for 5 days *in vitro* in the presence of BDNF, CNTF, or GDNF and stained against transmembrane protein APP (green) and Ca_v_2.2 (magenta) (scale bar: 5 μm). With CNTF or GDNF Ca_v_2.2 signals were significantly reduced in comparison to BDNF (BDNF 1.00 ± 0.11, Q_2_ 1.00, *n* = 5, *N* = 130; CNTF 0.65 ± 0.05, Q_2_ 0.65, *n* = 6, *N* = 133; GDNF 0.47 ± 0.04, Q_2_ 0.47, *n* = 6, *N* = 145; p(B-C) = 0.0081, p(B-G) = 0.0002), particularly at axon terminal protrusions, whereas APP immunoreactivities were not decreased. Similar results were obtained by normalizing Ca_v_2.2 intensities against internal reference protein APP. **(B)** In comparison to BDNF-treated motoneurons CNTF- and GDNF-treated cells displayed reduced frequencies of spontaneous Ca^2+^ transients in their corresponding growth cones (BDNF 0.89 ± 0.11, Q_2_ 0.6, *N* = 75; CNTF 0.69 ± 0.13, Q_2_ 0.3, *N* = 77; GDNF 0.50 ± 0.08, Q_2_ 0.3, *N* = 73; p(B-C) = 0.0306, p(B-G) = 0.0078). (Right panel) Representative recordings of axonal growth cones of motoneurons cultured with BDNF (blue), CNTF (magenta) or GDNF (green) showing calcium spikes. **(C)** Motoneurons were cultured on laminin-221/211 for 7 days *in vitro* in the presence of BDNF, CNTF, or GDNF and stained against tau (scale bar: 150 μm). Both CNTF- (466.6 ± 35.41 μm, Q_2_ 465.6 μm, *n* = 6, *N* = 263) and GDNF-treated (454.8 ± 19.28 μm, Q_2_ 440.9 μm, *n* = 6, *N* = 200) motoneurons grew significantly longer axons (p(B-C) = 0.0256, p(B-G) = 0.0470) in comparison to BDNF-treated cells (356 ± 22.16 μm, Q_2_ 342 μm, *n* = 6, *N* = 275).

The observed excitability phenotype in embryonic *trkBTK*^−/−^ motoneurons raised the question whether BDNF is necessary to mediate Ca_v_2.2 clustering and calcium influx in axonal growth cones modulating axonal outgrowth on laminin-221/211. For this purpose we cultured wild type motoneurons in the presence of BDNF, CNTF or GDNF on laminin-221/211 for 5 days (Figure 2). CNTF- and GDNF-treated motoneurons developed growth cones with significantly decreased Ca_v_2.2 immunoreactivities in comparison to BDNF-treated cells, particularly at protrusions (Figure 2A). The transmembrane protein APP (amyloid precursor protein) was used as a reference control (Figure 2A). These defects in Ca_v_2.2 clustering were reflected by significantly reduced frequencies of local spontaneous calcium transients in the absence of BDNF (Figure 2B). Interestingly, the subtle difference between CNTF and GDNF in terms of Ca_v_2.2 accumulation also found expression in the determined frequency of calcium spikes (Figure 2B). In a next step, we cultured wild type motoneurons on laminin-221/211 for 7 days under the same experimental conditions determining axonal outgrowth (Figure 2C). Only in the presence of BDNF, motoneurons were able to recognize differentiation signals provided by β2-chain laminin displaying the lowest axon growth rate in comparison to CNTF- and GDNF-treated motoneurons (Figure 2C, right panel). Soma size, dendrite complexity and cell survival were comparable between each neurotrophic factor validating their biological activity and emphasizing a dominant axonal phenotype (Figures S2B–D).

### A transient BDNF pulse is sufficient to enhance Ca_v_2.2 clustering and calcium influx in growth cones of embryonic motoneurons cultured on laminin-221

**Figure 3:**
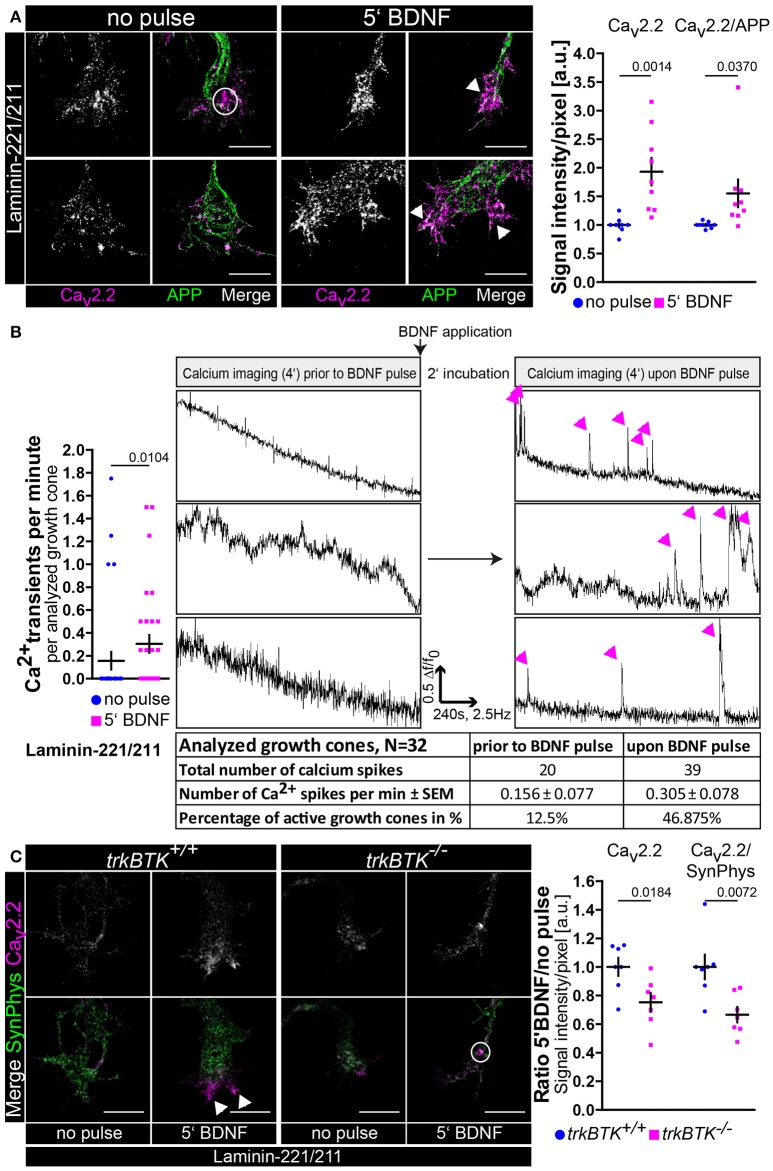
BDNF signaling is sufficient to induce Ca_v_2.2 clustering and local Ca^2+^ transients at growth cone protrusions of embryonic motoneurons on laminin-221. **(A)** Representative images of axonal growth cones of non-pulsed and BDNF-pulsed axonal growth cones on laminin-221/211 stained against Ca_v_2.2 (magenta) and APP (green) (scale bar: 5 μm). Upon BDNF pulse Ca_v_2.2 channels clustered at growth cone tips as highlighted by white arrowheads. In the non-pulsed condition potential accumulations of Ca_v_2.2 channels were rather detected in central growth cone areas as indicated by white circles. Ca_v_2.2 levels revealed a significant increase upon BDNF pulse (no pulse 1.00 ± 0.04, Q_2_ 1.00, *n* = 9, *N* = 221; 5′ BDNF 1.93 ± 0.24, Q_2_ 1.75, *n* = 9, *N* = 245; *p* = 0.0014). The ratio of Ca_v_2.2 and APP immunoreactivities yielded similar results. **(B)** These structural changes upon treatment with BDNF were matched by significantly enhanced frequencies of spontaneous Ca^2+^ transients in comparison to non-pulse controls (no pulse 0.16 ± 0.08, Q_2_ 0, IQR 0, *N* = 32; 5′ BDNF 0.30 ± 0.08, Q_2_ 0, IQR 2, *N* = 32; *p* = 0.0104). (Right panel) Identical growth cones were imaged prior to and 2 min after BDNF pulse. Representative recordings of non-pulsed and BDNF-pulsed traces showed increased numbers of Ca^2+^ spikes (magenta arrowheads) in response to BDNF. Upon BDNF the total number of calcium spikes almost doubled and the percentage of active growth cones displaying at least one spike per recording was greatly increased. **(C)** Representative images of non-pulsed and BDNF-pulsed *trkBTK*^+/+^ and *trkBTK*^−/−^ growth cones stained against Ca_v_2.2 (magenta) and synaptophysin (green) (scale bar: 5 μm). In wild type cells the acute application of BDNF resulted in increased Ca_v_2.2 immunoreactivity at growth cone protrusions (indicated by white arrowheads). This effect was not visible in *trkBTK*^−/−^ axonal growth cones, where Ca_v_2.2 immunoreactivity rather occurred in central growth cone regions as emphasized by white circles. Synaptophysin appeared comparable in each condition serving as internal reference protein. (Right panel) Statistical significance was determined by the ratio of BDNF-pulsed vs. non-pulsed growth cones of *trkBTK*^+/+^ and *trkBTK*^−/−^ motoneurons with respect to Ca_v_2.2 alone (*trkBTK*^+/+^ 1.00 ± 0.06, Q_2_ 1.00, *n* = 7, N(no pulse) = 88, N(5′ BDNF) = 102; *trkBTK*^−/−^ 0.75 ± 0.07, Q_2_ 0.79, *n* = 7, N(no pulse) = 100, N(5′ BDNF) = 106; *p* = 0.0184) and Ca_v_2.2 signal intensities normalized against synaptophysin highlighting the reduced responsiveness of *trkBTK*^−/−^ growth cones to acute BDNF application.

All the data reported up to now hypothesize that BDNF/trkB signaling is central for cellular processes in axons and growth cones of embryonic mouse motoneurons cultured on laminin-221/211 with respect to axon extension and neuronal excitability. These findings have been obtained by permanent exposure of BDNF to embryonic motoneurons for 5 or 7 days *in vitro*. In a next step we wanted to examine whether one single application of BDNF is sufficient to induce the aforementioned and discussed effects of BDNF in axonal growth cones. Thus, we cultured wild type motoneurons on laminin-221/211 for 4 days in the presence of BDNF and CNTF (Figure 3). Then, BDNF was deprived from the medium and cells were cultured for further 16 h only with CNTF. Finally, motoneurons were pulsed with BDNF for 5 min prior to fixation. Upon BDNF application Ca_v_2.2 immunoreactivity was significantly enhanced, again particularly at protrusions, which corresponds to enhanced Ca_v_2.2 clustering (Figure 3A). Notably, this alteration was not detectable when motoneurons were treated with acetone during the staining procedure indicating BDNF-induced Ca_v_2.2 clustering and surface expression since acetone dissolves the majority of phospholipid components (Figure S3A). Additionally, these observations were accompanied by increased frequencies of spontaneous calcium transients (Figure 3B). For this specific experiment the same axonal growth cones were imaged before and 2 min after BDNF application (Figure 3B, right panel). The total number of calcium spikes doubled, and the percentage of active growth cones almost quadrupled (Figure 3B, right panel). In line with these results, the responsiveness of *trkBTK*^−/−^ growth cones to acute BDNF treatment was significantly dampened in comparison to controls (Figure 3C). In trkB mutant growth cones there was no difference in Ca_v_2.2 signals between both conditions, whereas wild type controls exhibited enhanced Ca_v_2.2 clustering upon BDNF pulse (Figure 3C). The reference protein synaptophysin appeared not regulated, neither by genotype nor by BDNF stimulation (Figure 3C). In summary, these data support the working hypothesis that in axonal growth cones of embryonic mouse motoneurons BDNF/trkB signaling essentially contributes to laminin β2-chain-mediated neuronal excitability and axon growth.

### Mutual dependence of spontaneous Ca^2+^ transients and local actin cytoskeleton in growth cones of embryonic motoneurons

**Figure 4:**
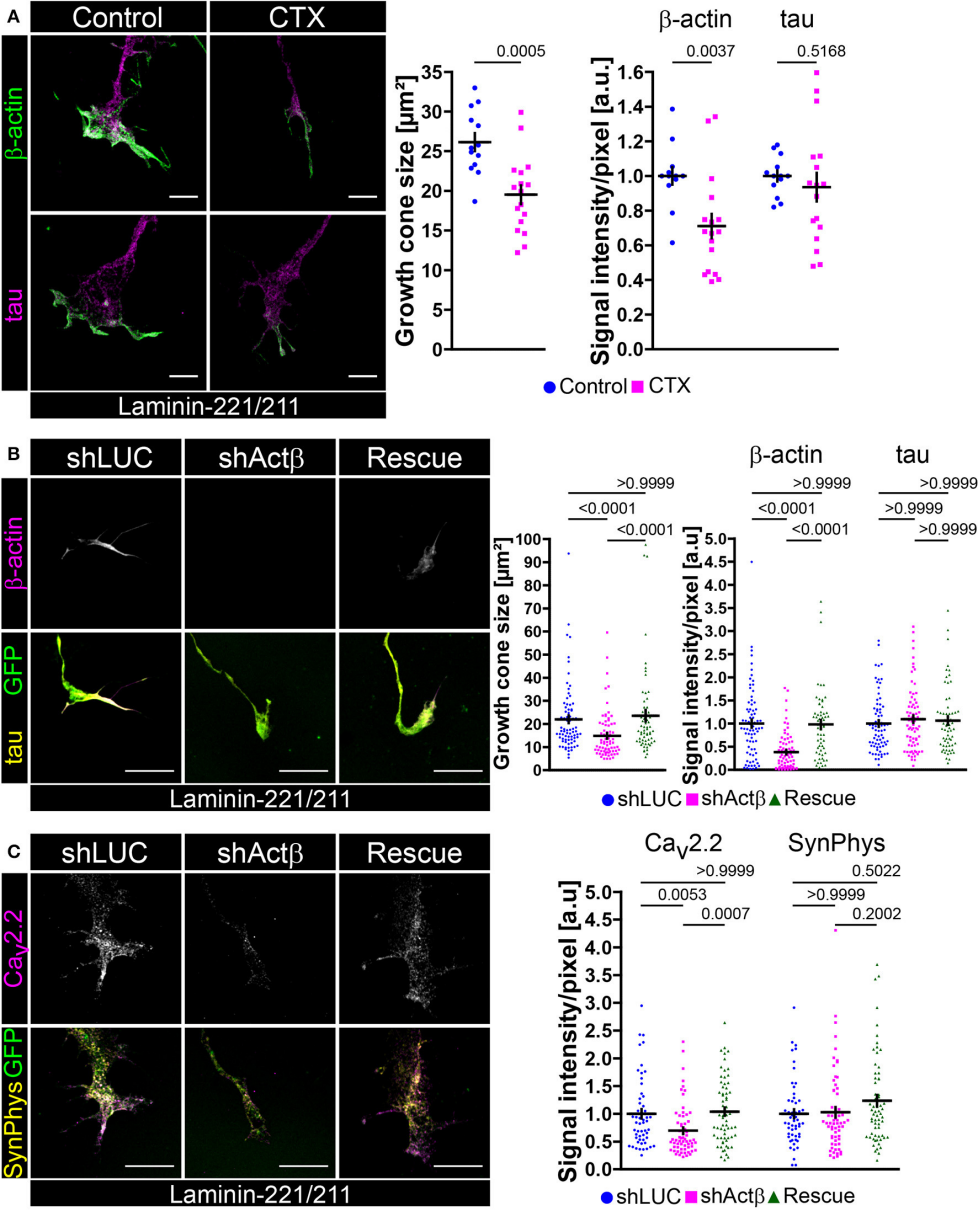
Mutual regulation of neuronal excitability and β-actin presence in axonal growth cones of embryonic motoneurons on laminin-221. **(A)** Representative images of growth cones of embryonic motoneurons cultured on laminin-221/211 for 5 days *in vitro* with or without 30 nM CTX and stained against β-actin (green) and tau (magenta) (scale bar: 5 μm). CTX-treated motoneurons developed smaller growth cones (Control 26.14 ± 1.13 μm^2^, Q_2_ 25.4 μm^2^, *n* = 13, *N* = 226; 30 nM CTX 19.51 ± 1.17 μm^2^, Q_2_ 19.96 μm^2^, *n* = 17, *N* = 274; *p* = 0.0005) with β-actin deficits (Control 1.00 ± 0.05, Q_2_ 1.00; 30 nM CTX 0.71 ± 0.07, Q_2_ 0.68; *p* = 0.0037). Tau levels were comparable between both conditions. **(B)** Representative images of axonal growth cones of β-actin depleted and control motoneurons cultured on laminin-221/211 for 5 days *in vitro* and stained against β-actin (magenta) and tau (yellow) (scale bar: 5 μm). Cells were selected by GFP expression (green) indicating successful lentiviral transduction. In comparison to shLUC-transduced control motoneurons β-actin shRNA-transduced cells revealed significantly smaller growth cones (shLUC 22.02 ± 1.68 μm^2^, Q_2_ 16.89 μm^2^, IQR 13.07 μm^2^, *N* = 76; shActβ 14.85 ± 1.18 μm^2^, Q_2_ 11.70 μm^2^, IQR 11.45 μm^2^, *N* = 74) and highly decreased β-actin signals (shLUC 1.00 ± 0.09, Q_2_ 0.90, IQR 0.92; shActβ 0.38 ± 0.05, Q_2_ 0.27, IQR 0.53). The microtubule cytoskeleton represented by tau appeared not affected. Re-expression of β-actin (“Rescue”) was able to rescue growth cone size (23.54 ± 2.44 μm^2^, Q_2_ 17.09 μm^2^, IQR 13.15 μm^2^, *N* = 62) and β-actin immunoreactivity (0.98 ± 0.09, Q_2_ 0.96, IQR 0.82) validating the obtained β-actin based growth cone phenotype. **(C)** Representative images of β-actin depleted growth cones on laminin-221/211 stained against Ca_v_2.2 (magenta) and synaptophysin (yellow) (scale bar: 5 μm). Knockdown of β-actin resulted in reduced Ca_v_2.2 signals (shLUC 1.00 ± 0.09, Q_2_ 0.84, IQR 0.75, *N* = 54; shActβ 0.70 ± 0.06, Q_2_ 0.52, IQR 0.46, *N* = 63), whereas synaptophysin levels were not altered. Re-entry of β-actin was able to rescue the detected phenotype (Rescue 1.04 ± 0.07, Q_2_ 1.05, IQR 0.90, *N* = 64).

Ca_v_2.2 clustering and local calcium signaling depends on BDNF/trkB signaling in axonal growth cones of embryonic motoneurons cultured on laminin-221/211. There is evidence that calcium and actin mutually interact with each other creating regulatory feedback loops (Robinson et al., 2010; Saneyoshi and Hayashi, 2012). Therefore, we wanted to examine how spontaneous Ca^2+^ influx mediated through Ca_v_2.2 modulates actin in growth cones of embryonic motoneurons and *vice versa*, how manipulation of the actin cytoskeleton affects Ca_v_2.2 accumulation (Figure 4). Embryonic motoneurons were cultured on laminin-221/211 for 5 days, treated with ω-conotoxin (30 nM) and stained against β-actin (Figure 4A), the most important actin isoform in axonal growth cones of embryonic motoneurons modulating growth cone size and morphology (Moradi et al., 2017). Treatment with CTX significantly reduced the size of axonal growth cones (Figure 4A). Furthermore, β-actin immunoreactivity was affected in these structures, whereas tau levels appeared comparable (Figure 4A). In turn, knockdown of β-actin by lentiviral shRNA in embryonic motoneurons cultured on laminin-221/211 for 5 days *in vitro* resulted in smaller growth cones and depletion of β-actin signal intensities (Figure 4B). These findings were caused by loss of β-actin since re-expression of this actin isoform was able to rescue the obtained phenotype (Figure 4B). Tau levels were not altered highlighting the specific manipulation of the actin cytoskeleton (Figure 4B). Intriguingly, β-actin knockdown also impaired local Ca_v_2.2 clustering in these cells, whereas synaptophysin immunoreactivity appeared similar in each condition (Figure 4C). Again, the re-expression of β-actin was sufficient to rescue the observed defects (Figure 4C) emphasizing the contribution of actin to the accumulation of Ca_v_2.2 in axonal growth cones of motoneurons cultured on laminin-221/211.

In order to further strengthen our working hypothesis of a mutual regulation of actin and calcium in axonal growth cones of motoneurons we pharmacologically manipulated the BDNF-mediated effect on Ca_v_2.2 clustering by treatment with either Src kinase inhibitor cocktail PP1 or cytochalasin D (CytD), respectively (Figures S3C,D). Mycotoxin cytochalasin D depolymerizes actin in hippocampal neurons *in vitro* (Bradke and Dotti, 1999) and it has been reported that Ca_v_2.2 calcium channels are located within lipid raft-associated microdomains which are sensitive to CytD (Robinson et al., 2010). Other studies have demonstrated that neurotrophic factor signaling asymmetrically activates Src kinases which in turn promotes actin mRNA translocation, local translation and Ca^2+^-dependent growth cone shaping (Hüttelmaier et al., 2005; Yao et al., 2006; Sasaki et al., 2010). Furthermore, Src kinases appear to phosphorylate profilin at Tyr129 promoting actin polymerization and Wnt/Ca^2+^ signaling (Fan et al., 2012; Frantzi et al., 2016). In line with these observations BDNF was not able to increase Ca_v_2.2 immunoreactivity in growth cones of embryonic motoneurons when these cells had been treated with either PP1 or CytD for 30 min prior to BDNF stimulation (Figure S3D). In summary, we provide experimental evidence that Ca_v_2.2 and β-actin signaling cascades mutually interact with each other in growth cones of motoneurons cultured on laminin-221.

### Defective actin cytoskeleton in axonal growth cones of *trkBTK^−/−^* and BDNF-deprived embryonic motoneurons cultured on laminin-221

**Figure 5:**
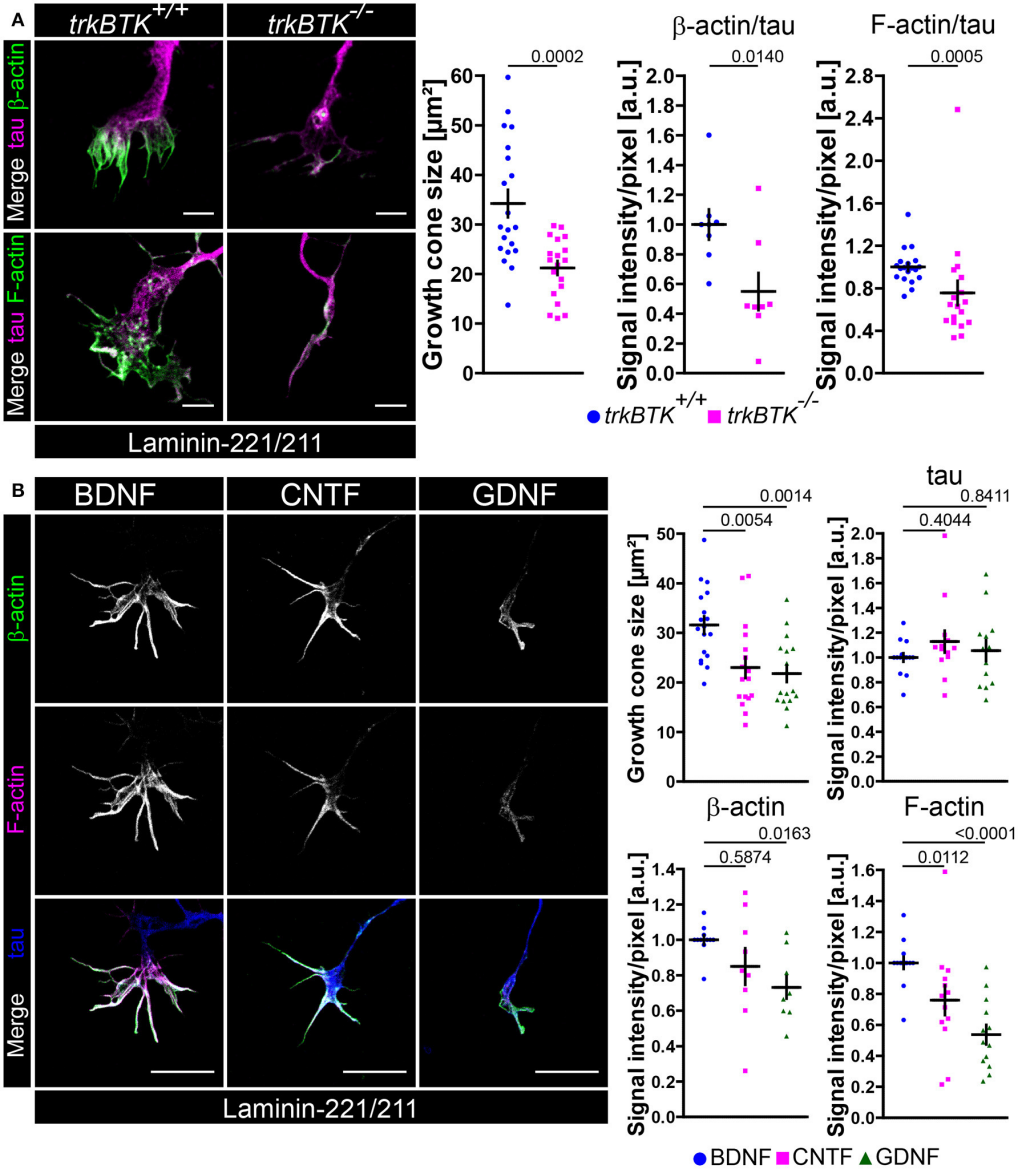
Actin cytoskeleton deficits in growth cones of *trkBTK*^−/−^ and BDNF-deprived embryonic motoneurons on laminin-221. **(A)** Representative images of growth cones of *trkBTK*^+/+^ and *trkBTK*^−/−^ motoneurons cultured on laminin-221/211 for 5 days *in vitro* and stained against tau (magenta) and either β-actin or F-actin (green) (scale bar: 5 μm). *TrkBTK*^−/−^ motoneurons developed significantly smaller growth cones (*trkBTK*^+/+^ 34.23 ± 2.77 μm^2^, Q_2_ 29.45 μm^2^, *n* = 20, *N* = 308; *trkBTK*^−/−^ 21.22 ± 1.41 μm^2^, Q_2_ 22.81 μm^2^, *n* = 19, *N* = 341; *p* = 0.0002) with β-actin (*trkBTK*^+/+^ 1.00 ± 0.10, Q_2_ 1.00, *n* = 8, *N* = 154; *trkBTK*^−/−^ 0.55 ± 0.12, Q_2_ 0.45, *n* = 8, *N* = 167; *p* = 0.0140) and F-actin (*trkBTK*^+/+^ 1.00 ± 0.04, Q_2_ 0.99, IQR 0.15, *n* = 19, *N* = 254; *trkBTK*^−/−^ 0.76 ± 0.11, Q_2_ 0.64, IQR 0.44, *n* = 18, *N* = 298; *p* = 0.0005) deficits in comparison to wild type controls. Tau levels were comparable between both genotypes. **(B)** Representative images of axonal growth cones of embryonic motoneurons cultured on laminin-221/211 for 5 days *in vitro* in the presence of BDNF, CNTF or GDNF and stained against β-actin (green), F-actin (magenta) and tau (blue) (scale bar: 5 μm). Growth cones of CNTF- and GDNF-treated motoneurons were significantly reduced in size (BDNF 31.59 ± 1.75 μm^2^, Q_2_ 31.05 μm^2^, *n* = 18, *N* = 385; CNTF 23.01 ± 2.12 μm^2^, Q_2_ 20.73 μm^2^, *n* = 17, *N* = 425; GDNF 21.79 ± 1.69 μm^2^, Q_2_ 18.27 μm^2^, *n* = 17, *N* = 435; p(B-C) = 0.0054, p(B-G) = 0.0014) showing F-actin deficits (BDNF 1.00 ± 0.04, Q_2_ 1.00, IQR 0.01, *n* = 14, *N* = 276; CNTF 0.76 ± 0.10, Q_2_ 0.79, IQR 0.33, *n* = 13, *N* = 308; GDNF 0.54 ± 0.06, Q_2_ 0.49, IQR 0.37, *n* = 13, *N* = 314; p(B-C) = 0.0112, p(B-G) < 0.0001). GDNF-treated growth cones revealed a reduction in β-actin levels. Tau was comparable in each analyzed condition.

So far, we have demonstrated that Ca_v_2.2 accumulation and spontaneous Ca^2+^ influx through VGCCs are disturbed on laminin-221 when BDNF/trkB signaling is impaired. Furthermore, it appears that local Ca^2+^ elevations have an impact on the local actin cytoskeleton in axonal growth cones of embryonic motoneurons. In turn, actin appears to contribute to the positioning of Ca_v_2.2 calcium channels, a prerequisite for laminin β2-chain induced growth cone formation processes. Thus, we wanted to investigate the actin cytoskeleton in growth cones of *trkBTK*^−/−^ embryonic motoneurons cultured on laminin-221/211 for 5 days, a time point where defects in Ca_v_2.2 accumulation and Ca^2+^ signaling were detectable (Figure 5A). *TrkBTK*^−/−^ motoneurons developed significantly smaller growth cones in comparison to wild type controls (Figure 5A). This finding was accompanied by significantly decreased β-actin and F-actin intensities, whereas the microtubule stabilizing tau protein appeared not affected (Figure 5A). Since BDNF has a strong impact on axon growth on laminin-221/211 (Figure 2C) we also examined the actin cytoskeleton in growth cones of wild type motoneurons in the presence of BDNF, CNTF or GDNF (Figure 5B). In comparison to BDNF-treated motoneurons, CNTF- and GDNF-treated cells developed significantly smaller growth cones with deficits in F-actin, but not in tau protein levels (Figure 5B). In the case of GDNF there was also a significant reduction in β-actin immunoreactivity (Figure 5B). The impact of BDNF/trkB signaling on the presynaptic compartment, particularly on the actin cytoskeleton, raised the question whether this signaling pathway might also control the recruitment and translocation of actin mRNAs to these specific sites. Thus, we performed fluorescence *in situ* hybridization with 3′ biotinylated oligonucleotides against highly conserved coding regions of actin mRNA (Rossoll et al., 2003; Jablonka et al., 2007; Figures S4A–C). Upon BDNF pulse we observed increased actin mRNA signal intensities in growth cones of embryonic motoneurons cultured on laminin-221/211 for 5 days in comparison to non-pulsed controls (Figure S4A), which is in agreement with previous reports (Willis et al., 2007; Sasaki et al., 2010). Furthermore, we detected a significant reduction of actin mRNA immunoreactivity in *trkBTK*^−/−^ growth cones (Figure S4B), as well as in axonal growth cones of motoneurons cultured only with CNTF or GDNF (Figure S4C). Axon extension, axon guidance and growth cone formation during neural development require coordinated actions of actin and microtubules. To further validate the actin phenotype in growth cones of *trkBTK*^−/−^ and BDNF-deprived embryonic motoneurons we tested additional microtubule markers, i.e., glutamylated and tyrosinated tubulin, detecting no apparent differences between each genotype and condition (Figures S4D,E). Even though BDNF and GDNF both activate tyrosine kinases, i.e., trkB and c-ret, only BDNF appears to sustain Ca_v_2.2 clustering and F-actin assembly in growth cones. Thus, we wanted to investigate whether the intracellular distribution of trkB and c-ret diverges from each other, especially in these presynaptic structures (Figure S1D). *De facto*, trkB appeared enriched in protrusions, whereas c-ret signals appeared highest in rather central growth cone regions (Figure S1D).

### On laminin-111 the withdrawal of BDNF causes no axonal phenotype in embryonic motoneurons

**Figure 6:**
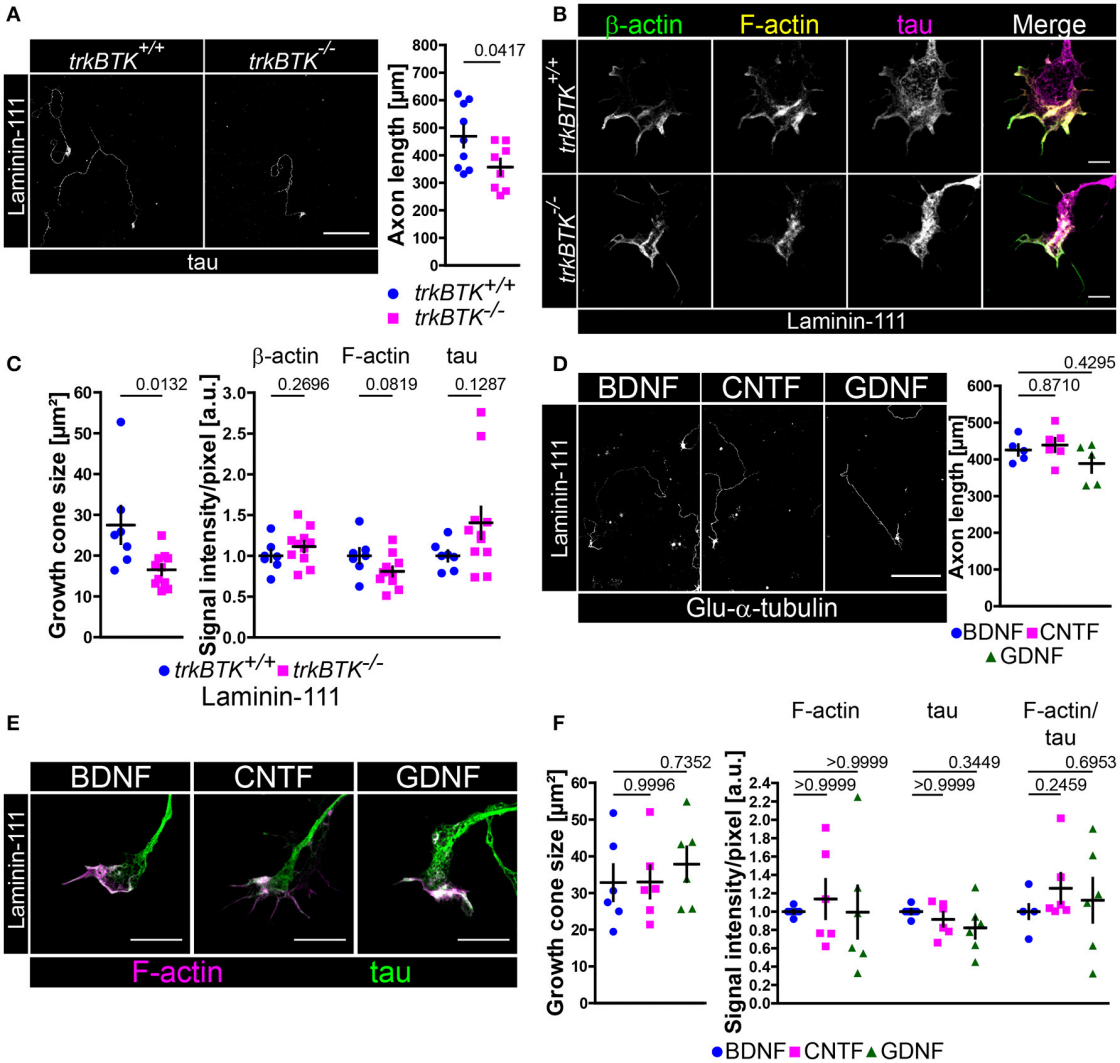
On laminin-111 BDNF is dispensable for axon growth and F-actin assembly in growth cones. **(A)** Representative images of *trkBTK*^+/+^ and *trkBTK*^−/−^ motoneurons cultured on laminin-111 for 7 days *in vitro* and stained against tau (scale bar: 150 μm). TrkB mutant motoneurons (357.2 ± 29.33 μm, Q_2_ 363 μm, *n* = 8, *N* = 579) grew shorter axons (*p* = 0.0417) in comparison to wild type controls (468.8 ± 39.31 μm, Q_2_ 454.9 μm, *n* = 9, *N* = 615). **(B)** Representative images of axonal growth cones of *trkBTK*^+/+^ and *trkBTK*^−/−^ motoneurons cultured for 5 days *in vitro* on laminin-111 and stained against β-actin (green), F-actin (yellow), and tau (magenta) (scale bar: 5 μm). **(C)** *TrkBTK*^−/−^ motoneurons (16.55 ± 1.26 μm^2^, Q_2_ 17.12 μm^2^, *n* = 11, *N* = 178) developed significantly smaller growth cones (*p* = 0.0132) than the corresponding controls (27.51 ± 4.58 μm^2^, Q_2_ 25.06 μm^2^, *n* = 7, *N* = 113). There were no significant alterations between both genotypes with respect to β-actin, F-actin and tau levels. **(D)** Representative images of wild type motoneurons cultured on laminin-111 for 7 days *in vitro* with BDNF, CNTF or GDNF and stained against tau (scale bar: 150 μm). There was no difference in axonal elongation between each neurotrophic factor (BDNF 425.3 ± 14.93 μm, Q_2_ 423.3 μm, *n* = 5, *N* = 256; CNTF 439.0 ± 18.37 μm, Q_2_ 439.9 μm, *n* = 6, *N* = 258; GDNF 388.6 ± 24.47 μm, Q_2_ 412.1 μm, *n* = 5, *N* = 273; p(B-C) = 0.8710, p(B-G) = 0.4295). **(E)** Representative images of axonal growth cones of motoneurons cultured on laminin-111 for 5 days *in vitro* in the presence of BDNF, CNTF or GDNF and stained against F-actin (magenta) and tau (green) (scale bar: 5 μm). **(F)** There was no significant difference in growth cone size between each condition (BDNF 32.83 ± 4.93 μm^2^, Q_2_ 29.04 μm^2^, *n* = 6, *N* = 165; CNTF 33.01 ± 4.41 μm^2^, Q_2_ 30.96 μm^2^, *n* = 6, *N* = 175; GDNF 37.85 ± 4.72 μm^2^, Q_2_ 38.52 μm^2^, *n* = 6, *N* = 172; p(B-C) = 0.9996, p(B-G) = 0.7352). Furthermore, F-actin (BDNF 1.00 ± 0.02, Q_2_ 1.00, IQR 0.04, *n* = 6, *N* = 165; CNTF 1.14 ± 0.22, Q_2_ 0.95, IQR 0.97, *n* = 6, *N* = 175; GDNF 0.99 ± 0.28, Q_2_ 0.79, IQR 1.02, *n* = 6, *N* = 172; p(B-C) > 0.9999, p(B-G) > 0.9999) and tau levels appeared not altered.

There are different laminin isoforms exerting various functions. Laminin-221 which contains the aforementioned β2-chain plays an important role at the synaptic cleft of neuromuscular junctions (Noakes et al., 1995), whereas β1-chain laminins are important for Schwann cell differentiation (Colognato and Yurchenco, 2000). So far, differences in axon extension (Figure 1D) and β-actin/F-actin presence (Figure 5A, Figure S4F) have been observed in axonal growth cones of trkB-deficient motoneurons cultured on laminin-221/211 without affecting soma size and dendrite complexity (Figure S2A). In order to further characterize and emphasize the collaborative effort of BDNF/trkB signaling and β2-chain laminins we cultured *trkBTK*^−/−^ motoneurons on laminin-111 which carries the β1-chain (Figures 6A–C). In comparison to *trkBTK*^+/+^ cells trkB-deficient motoneurons grew shorter axons (Figure 6A) and developed smaller growth cones (Figure 6B). However, these smaller growth cones revealed no alterations in β-actin and F-actin levels (Figures 6B,C, Figure S4F). Soma size and dendrite morphology were comparable between both genotypes (Figure S2E). Similar to the results obtained with Ca_v_2.2 (Figure S1C) the use of laminin-221/211 as matrix protein seemed to result in enhanced F-actin levels in axonal growth cones of control motoneurons in comparison to laminin-111, whereas this difference was not detectable in *trkBTK*^−/−^ growth cones (Figure S4F).

Since trkB deficiency leads to tremendous developmental defects and early death right after birth (Klein et al., 1993) and since we cannot exclude “off-target” effects during embryonic development which might influence the observed morphological abnormalities in trkB-deficient motoneurons cultured on laminin-111 with respect to axon growth and growth cone size we went on to culture wild type motoneurons on laminin-111 in the presence of BDNF, CNTF or GDNF (Figures 6D–F). In this way, normal embryonic motoneuron development is ensured and the contribution of BDNF and laminin signaling to axon extension and growth cone formation can be addressed experimentally. After 7 days in culture we observed no significant difference in axon growth (Figure 6D). Soma size and dendrite complexity were also comparable in each condition (Figure S2F). After 5 days in culture BDNF-, CNTF-, and GDNF-treated motoneurons developed growth cones with comparable sizes (Figures 6E,F). F-actin and tau signal intensities appeared also not altered (Figures 6E,F). We concluded from these data that the combined efforts of BDNF/trkB and laminin β2-chain signaling are crucial for growth cone formation in embryonic motoneurons in terms of Ca_v_2.2 clustering and local calcium signaling, as well as actin polymerization or stabilization, respectively. On laminin-111 BDNF appears dispensable.

### A transient BDNF pulse is sufficient to induce phosphorylation of LIM kinases and cofilin which leads to F-actin assembly in axonal growth cones of embryonic motoneurons cultured on laminin-221

**Figure 7:**
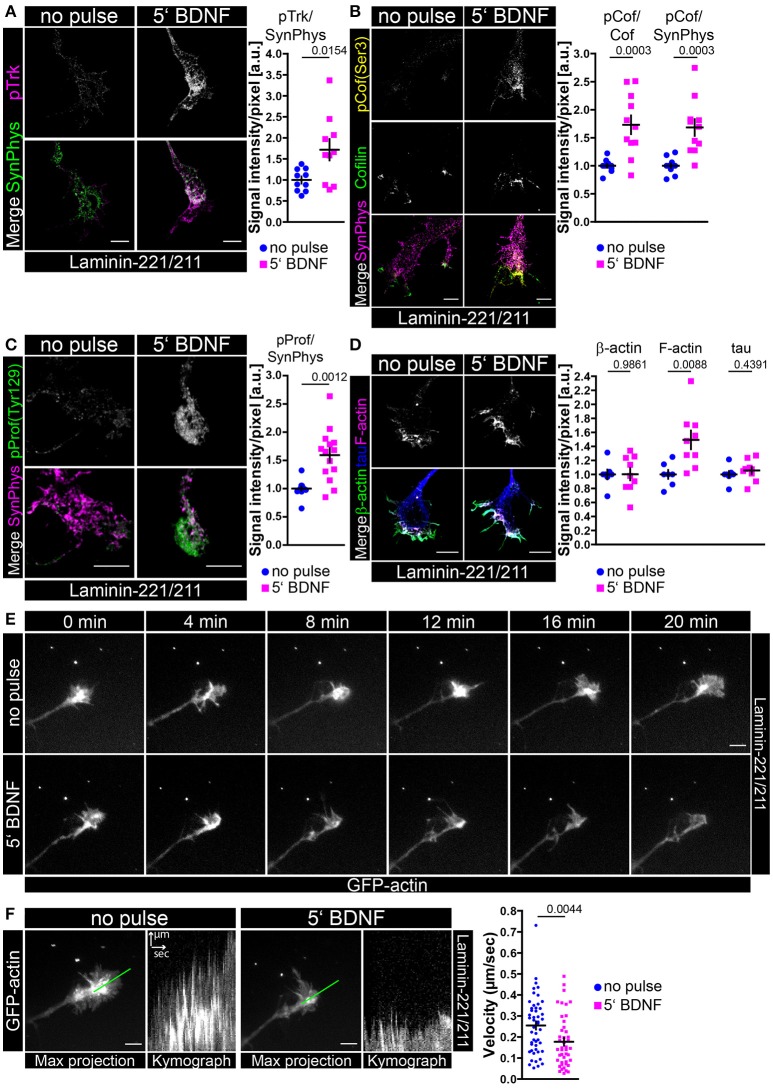
Transient BDNF application on laminin-221 activates trkB signaling pathways in growth cones of motor axons resulting in phosphorylation of cofilin and profilin stabilizing actin. **(A)** Representative images of axonal growth cones of embryonic motoneurons cultured on laminin-221/211 for 5 days *in vitro* and stained against phospho-trk (magenta) and synaptophysin (green) (scale bar: 5 μm). Upon BDNF pulse phospho-trk immunoreactivity was significantly increased (no pulse 1.00 ± 0.08, Q_2_ 1.00, *n* = 10, *N* = 136; 5′ BDNF 1.72 ± 0.25, Q_2_ 1.63, *n* = 10, *N* = 137; *p* = 0.0154), whereas synaptophysin levels were comparable. **(B)** BDNF stimulation also increased phospho-cofilin (Ser 3, yellow) intensities (pCof/cofilin–no pulse 1.00 ± 0.03, Q_2_ 1.00, *n* = 11, *N* = 174; 5′ BDNF 1.73 ± 0.17, Q_2_ 1.69, *n* = 11, *N* = 176; *p* = 0.0003), but did alter neither cofilin (green) nor synaptophysin (magenta) (scale bar: 5 μm). **(C)** Representative images of axonal growth cones stained against phospho-profilin (Tyr 129, green) and synaptophysin (magenta) (scale bar: 5 μm). Upon BDNF treatment the levels of phospho-profilin normalized to synaptophysin were enhanced (no pulse 1.00 ± 0.04, Q_2_ 1.00, IQR 0.09, *n* = 13, *N* = 200; 5′ BDNF 1.59 ± 0.12, Q_2_ 1.67, IQR 0.57, *N* = 14, *n* = 226; *p* = 0.0012). **(D)** In BDNF-pulsed growth cones F-actin (magenta) was significantly increased (1.49 ± 0.13, Q_2_ 1.48, *n* = 9, *N* = 236; *p* = 0.0088) in comparison to non-pulsed controls (1.00 ± 0.06, Q_2_ 1.00, *n* = 7, *N* = 194), whereas total β-actin (green) and tau (blue) appeared not changed (scale bar: 5 μm). **(E)** Live-cell imaging of axonal growth cones of embryonic motoneurons cultured for 5 or 6 days *in vitro* and transduced with lentiviral constructs expressing actin-GFP. Time-lapse series of the same growth cone prior to and after BDNF pulse (scale bar: 5 μm). Upon BDNF application filopodia dynamics appeared reduced indicating actin stabilization. **(F)** Filopodia dynamics were assessed as multiple kymographs revealing significantly reduced actin movement upon BDNF pulse (no pulse 0.25 ± 0.02 μm/s, Q_2_ 0.27 μm/s, IQR 0.20 μm/s, *N* = 49; 5′ BDNF 0.18 ± 0.02 μm/s, Q_2_ 0.14 μm/s IQR 0.16 μm/s, *N* = 44; *p* = 0.0044).

Up to now, we have gathered experimental evidence that BDNF/trkB signaling together with β2-chain laminin promotes spontaneous Ca^2+^ influx that corresponds to growth cone formation by enhanced F-actin assembly. In order to verify whether a transient BDNF pulse is sufficient to induce signaling pathways leading ultimately to actin stabilization we stimulated BDNF-deprived motoneurons with BDNF prior to fixation or protein lysis (Figure 7, Figure S5). Upon BDNF pulse phospho-trk immunoreactivity was significantly enhanced in axonal growth cones in comparison to non-pulsed controls, whereas synaptophysin levels were not altered (Figure 7A). We also detected a moderate trkB increase at growth cone protrusions (Figure S5A) which may relate with the aforementioned accumulation of Ca_v_2.2 channels upon BDNF application at the same structures (Figure 3, Figure S3). The upregulation of phospho-trk was also accompanied by increased intensities of phospho-cofilin (Ser 3) (Figure 7B) leading to the inactivation of cofilin. Total cofilin and synaptophysin served as internal reference proteins (Figure 7B). These results were reproducible on a biochemical level by probing whole motoneuron cell lysates with antibodies against phospho-trk, phospho-AKT, phospho-MAPK and phospho-cofilin (Figure S5C). TrkB activation corresponded to phosphorylation of AKT and MAPK, as well as cofilin (Figure S5C). Recent reports have demonstrated that trkB receptors interact with LIM kinase 1 and that BDNF/trkB signaling induces LIM kinase 1 phosphorylation (Sarmiere and Bamburg, 2004; Dong et al., 2012; Saito et al., 2013). We also detected increased phosphorylation of LIM kinase 1 and 2 after transient stimulation with BDNF (Figure S5C). Besides the inactivation of cofilin, phosphorylation of profilin at tyrosine 129 (Tyr 129) directly enhances actin polymerization. Upon BDNF pulse the levels of phospho-profilin (Tyr 129) were significantly increased in axonal growth cones in comparison to non-pulsed controls (Figure 7C). The obtained changes in phospho-profilin (Figure 7C) and phospho-cofilin (Figure 7B) were also reflected and validated by increased F-actin staining, whereas tau and β-actin levels were comparable between both conditions (Figure 7D). Additionally, the acute application of BDNF resulted in reduced actin movement in growth cones of motoneurons cultured on laminin-221/211 (Figures 7E,F, Movies S1, S2), which corresponded to an apparent shift from globular (G-actin) to filamentous β-actin (F-actin) (Figure S5D). On laminin-111, transient application of BDNF and moderate trkB phosphorylation (Figure S5E) appeared to increase β-actin immunoreactivity in axonal growth cones, whereas F-actin levels appeared not changed (Figure S5F). These results are in contrast to laminin-221/211, where β-actin appears not regulated upon BDNF pulse, whereas F-actin is significantly increased (Figure 7D, Figure S5D). These data strengthen the hypothesis that BDNF stabilizes the local actin cytoskeleton through F-actin assembly, in particular when motoneurons are cultured on laminin-221/211.

## Discussion

The impact of neurotrophic factors on motoneuron survival is widely accepted. However, the specific and differential role of distinct neurotrophic factors from different families such as BDNF (neurotrophins), CNTF (interleukin), and GDNF (GFLs) with respect to axon extension and growth cone formation in embryonic motoneurons is still unaddressed. In particular the orchestration of F-actin assembly and the precise placement of receptors and ion channels like Ca_v_2.2 at growth cones demand a better understanding. Here, we show that BDNF, but not CNTF or GDNF, regulates axonal growth cone formation through induction of calcium influx and actin stabilization in collaboration with β2-chain laminin signaling. In addition to the more biologically relevant calcium imaging approaches, axon length measurements represent a rather indirect but reliable readout for neuronal excitability, especially when motoneurons are cultured on β2-chain laminins. This re-growth potential of motor axons in particular can also be used to characterize changes in the actin cytoskeleton.

Several reports have shown that neurotrophic factors exert distinct subcellular functions in developmental processes promoting survival and/or differentiation of individual neuronal populations albeit activating similar signaling cascades. CNTF expression is barely detectable during development proposing that CNTF most notably promotes motoneuron survival and maintenance at postnatal and adult stages (Masu et al., 1993; Sendtner et al., 1994). However, soluble receptor cytokine-like factor 1 (CLF) and cardiotrophin-like cytokine (CLC) represent another ligand for CNTFR, in particular during development, also conveying motoneuron survival via gp130, LIFR and STAT-3 signaling pathways (Elson et al., 2000; Forger et al., 2003). In corticospinal motoneurons, BDNF mediates axonal arborization and branching, whereas IGF-1 and CNTF induce axon growth (Ozdinler and Macklis, 2006; Richter and Roskams, 2009). In line with these observations, application of CNTF or IGF-1 ameliorated the ALS-afflicted *pmn* phenotype (Sendtner et al., 1992; Bommel et al., 2002; Jablonka et al., 2011; Selvaraj et al., 2012). In comparison to CNTF and GDNF, NT-4 and BDNF displayed the most substantial response on ChAT activity in motoneurons (Kato and Lindsay, 1994; Zurn et al., 1996). Administration of GDNF to the spinal cord of ALS-afflicted mice opposes motoneuron degeneration, whereas application of GDNF to the muscle results in preservation of motoneuron function, indicating local responsibilities of GDNF (Suzuki et al., 2007, 2008). Expression of c-ret appears stable in postnatal motoneurons, whereas GDNFRα1 and 2 are downregulated after birth (Zhang and Huang, 2006). In contrast, the proportion of full-length trkB to truncated trkB is 2:1 during embryonic development, whereas this ratio becomes the opposite in postnatal life through protein cleavage and RNA splicing processes (Zhang and Huang, 2006).

Thus, there seem to be spatial and temporal differences between GDNF and BDNF during embryonic development. GDNF and BDNF both act in parts through the MAP kinase pathway including Erk1/2 as a positive regulator of neuronal differentiation, but only BDNF conveyed sustained phosphorylation of MAPK in mesencephalic neurons, whereas GDNF caused a rather transient MAPK phosphorylation (Feng et al., 1999a,b). Interestingly, sustained activation of MAPK resulted in differentiation, while transient MAPK signaling contributed to cell survival and proliferation (York et al., 1998; Kao et al., 2001). In this context, it has been reported that trkB recycling which depends upon kinase activity is crucial for sustained ERK1/2 signaling (Huang et al., 2009) and that trk receptors in endosomes or in the plasma membrane induce sustained or transient ERK1/2 activation, respectively (York et al., 1998; Mochizuki et al., 2001; Wu et al., 2001). Notably, *trkBTK*^−/−^ mice are lacking this kinase domain (Klein et al., 1993). In line with these observations we found a distinct distribution of GDNF receptor c-ret and BDNF receptor trkB at nerve terminals of embryonic motoneurons cultured on laminin-221/211 (Figure S1D). TrkB is localized at growth cone protrusions in close neighborhood to Ca_v_2.2 calcium channels, whereas c-ret is translocated in rather central growth cone regions. It is tempting to speculate that this spatial difference could account for the axonal phenotype in BDNF-deprived motoneurons although both neurotrophic factors activate tyrosine kinase signaling pathways.

### BDNF/trkB induces spontaneous Ca^2+^ transients in primary motoneurons

In the peripheral and central nervous system BDNF modulates cell survival, but above all synaptic function and neuronal morphology (Blum and Konnerth, 2005; Minichiello, 2009; Rauskolb et al., 2010; Park and Poo, 2013; Sasi et al., 2017). In embryonic motoneurons BDNF/trkB signaling promotes survival. However, it is not fully understood whether BDNF/trkB is also necessary for cellular differentiation such as axon elongation and growth cone maturation. *In vivo*, growth cones of motoneurons ultimately mature to the presynaptic compartment of neuromuscular junctions (NMJ). Mice deficient for the β2-chain develop a NMJ phenotype (Noakes et al., 1995), whereas β1-chain laminin isoforms seem to play a pivotal role in Schwann cell differentiation (Chen and Strickland, 2003; Yang et al., 2005; Yu et al., 2005). A direct comparison of β1- and β2-chain function on a cellular level appears restricted and elusive due to their specific functions in distinct cell types at different developmental stages. Thus, we wanted to study motoneuron differentiation in the presence of a β2-chain carrying laminin isoform to investigate in detail which neurotrophic factor promotes the formation of axonal growth cones in this defined context. In this study we used the β2-chain laminin isoform 221 due to its role in presynaptic maturation and maintenance (Noakes et al., 1995; Jablonka et al., 2007). Our data discover that in embryonic motoneurons cultured on this synapse-specific laminin isoform, BDNF application and signal transduction via its high affinity receptor trkB leads to induction of spontaneous Ca^2+^ transients in axonal growth cones which in turn corresponds to regulation of axon elongation. These enhanced spontaneous Ca^2+^ transients arise and originate from local Ca_v_2.2 clustering at growth cone protrusions (see Figures 1–3; Jablonka et al., 2007), which is mediated through the interaction of the pore-forming subunit (Ca_v_) of Ca_v_2.2 and the β2-chain of laminin-221 (Nishimune et al., 2004). Finally, the resulting calcium influx is recognized as an axonal signal resulting in restrained axon growth on laminin-221 (Jablonka et al., 2007). Those enhanced Ca^2+^ transients localized at axonal growth cones are not detectable on laminins carrying a β1-chain like laminin-111 (Jablonka et al., 2007). In fact, we observed reduced Ca_v_2.2 clustering in axonal growth cones of control motoneurons cultured on laminin-111 in direct comparison to laminin-221/211 (see Figure S1C). Therefore, we suggest that experiments to investigate cellular mechanisms corresponding to calcium channel accumulation and local excitability particularly in growth cones of primary cultured motoneurons should be conducted with β2-chain laminin isoforms such as laminin-221, likely in comparison with β1-chain laminin isoforms, e.g., laminin-111.

At mouse motor nerve terminals, P/Q-type (Ca_v_2.1) voltage-gated calcium channels are the main channel involved in exocytosis at NMJ release sites after development (Katz et al., 1997; Rosato Siri and Uchitel, 1999; Santafe et al., 2001). We know from studies with Ca_v_2.1 knockout mice that these mice suffer from neuromuscular endplate degeneration three weeks after birth (Nishimune et al., 2004). Thus, based on our study, it is tempting to speculate that BDNF/trkB signaling might play a role in motoneuron differentiation, maintenance and function. In this context, the importance of trkB signaling has already been shown in the *trkBTK*^−/−^ knockout mouse (Klein et al., 1993). These mice carry a truncated trkB receptor (gp95_trkB_) with a disabled kinase activity (Klein et al., 1993). *TrkBTK*^−/−^ mice die around birth exhibiting sensory and motor defects (Klein et al., 1993). Furthermore, experiments with adenovirus-mediated somatic gene transfer could demonstrate that post-synaptic AChR regions at motor endplates disassembled when trkB signaling was affected by overexpression of truncated trkB (Gonzalez et al., 1999). In addition, experiments from Xenopus nerve-muscle co-cultures clearly illustrated that trkB regulates synapse elimination at neuromuscular junctions through conversion of proBDNF to mature BDNF (Je et al., 2012). Similar symptoms regarding Ca_v_2.2 clustering, growth cone formation and defects in neurotransmission at neuromuscular synapses were observed in mouse models for spinal muscular atrophy (Kong et al., 2009; Park et al., 2010; Ruiz et al., 2010; Tejero et al., 2016; Jablonka and Sendtner, 2017). With respect to reduced spontaneous Ca^2+^ transients, affected Ca_v_2.2 accumulation and dysregulated axon growth on laminin-221/211 and laminin-111, *trkBTK*^−/−^ motoneurons resemble the *in vitro* phenotype of Smn-deficient embryonic motoneurons (Rossoll et al., 2003; Jablonka et al., 2007, 2014). The importance of cellular excitability for motoneuron survival and function is strengthened by recent reports on the voltage-gated sodium channel Na_v_1.9 which significantly contributes to motoneuron axon growth on laminin-111 (Subramanian et al., 2012; Wetzel et al., 2013) and the loss of the potassium channels K_v_2.1 which corresponds to motoneuron degeneration in SMA mouse models (Fletcher et al., 2017). Furthermore, neurotrophic factors like NGF and NT-3 differentially modulate Na_v_1.9 and Na_v_1.8 expression (Wilson-Gerwing et al., 2008), and BDNF signaling appears to coincide with Na_v_1.9-executed depolarization (Blum et al., 2002). Therefore, BDNF/trkB signaling might contribute to dysregulated cellular excitability which contributes to motoneuron disease.

### BDNF/trkB supports growth cone formation

Our study elucidates that β-actin presence at growth cone protrusions controls growth cone size and Ca_v_2.2 accumulation. *Vice versa*, local calcium transients modulate β-actin level and growth cone morphology. This mutual dependence appears important for differentiation of presynaptic structures. We know from several reports that BDNF/trkB signaling promotes phosphorylation of LIM kinase 1 and cofilin which results in actin polymerization and axonal outgrowth in hippocampal neurons (Sarmiere and Bamburg, 2004; Dong et al., 2012; Saito et al., 2013). We also observed a BDNF-induced phosphorylation of LIM kinases and cofilin in embryonic motoneurons which corresponded to reduced actin movement at growth cone protrusions indicating F-actin assembly and stabilization (Figure 7F, Figure S5D). LIM kinases phosphorylate cofilin proteins at serine 3 inactivating their function in actin depolymerization and rapid turnover of actin filaments (Moriyama et al., 1996; Mizuno, 2013; Ohashi, 2015). Interestingly, this actin stabilization in combination with Ca_v_2.2-β2-chain-evoked axonal differentiation signals resulted in restricted axon growth on laminin-221 in the presence of BDNF. CNTF- and GDNF-treated motoneurons developed smaller growth cones and grew longer axons on laminin-221 in comparison to BDNF-treated cells. On laminin-111 BDNF appeared not essential for axon extension and growth cone formation (Figures 6D–F). Furthermore, *TrkBTK*^−/−^ motoneurons revealed no apparent actin phenotype on laminin-111 despite developing growth cones smaller in size (Figures 6B,C). This further strengthens the important role of BDNF/trkB signaling in LIM kinase/cofilin-mediated F-actin assembly and the generation of local Ca^2+^ transients via Ca_v_2.2 accumulation when motoneurons are cultured on β2-chain laminin-221. These observations paralleled those from the central nervous system (CNS). Inactivation of cofilin in the CNS caused F-actin barbed ends and in turn resulted in enlargement of spines associated with enhanced AMPA receptor insertion (Gu et al., 2010). We also detected increased phosphorylation of profilin at Tyr 129 in growth cones upon BDNF stimulation leading to the assembly of G-actin into F-actin (Kang et al., 1999; Figure 7). Profilin contributes to neuronal development and synaptic plasticity by regulating cytoskeletal integrity and actin dynamics and by binding to piccolo, gephyrin, and drebrin among others (Wang et al., 1999; Fenster et al., 2000, 2003; Kim et al., 2003; Pilo Boyl et al., 2007; Waites et al., 2011; Görlich et al., 2012; Kullmann et al., 2012; Michaelsen-Preusse et al., 2016). There are also implications for profilin in Wnt/Ca^2+^ signaling (Frantzi et al., 2016) pointing to the aforementioned mutual dependence of Ca^2+^ transients and actin dynamics in axonal growth cones. It has been reported that VEGF (vascular endothelial growth factor) and Src kinases induce phosphorylation of profilin at Tyr 129 enhancing actin polymerization particularly at peripheral structures (Fan et al., 2012; Pronto-Laborinho et al., 2014). In agreement with these observations BDNF was not able to enhance Ca_v_2.2 clustering in growth cones of motoneurons upon inhibition of Src kinases (Figures S3C,D), highlighting the mutual dependence of actin and calcium signaling. Src kinases represent important downstream mediators of neurotrophic factor signaling recruiting β-actin transcripts to the periphery, stimulating local protein synthesis and mediating growth cone turning in a calcium-dependent manner (Zhang et al., 1999, 2001; Hüttelmaier et al., 2005; Yao et al., 2006; Willis et al., 2007; Sasaki et al., 2010). Recently, profilin mutations have been identified in sporadic and familial ALS cases possibly affecting the actin-binding domain and dysregulating actin polymerization, making this protein clinically relevant (Wu et al., 2012; Chen et al., 2013; Ingre et al., 2013; Del Poggetto et al., 2015, 2016; Freischmidt et al., 2015; Smith et al., 2015). Interestingly, mutant profilin is localized in cytoplasmic aggregates also comprising TDP43, ubiquitin and p62 (Wu et al., 2012; Tanaka et al., 2016).

Taken together, it is still an open question how BDNF/trkB signal transduction mechanistically leads to Ca_v_2.2 accumulation. A direct interaction between both molecules is not known so far although the potential localization in close proximity particularly at growth cone protrusions of embryonic motoneurons raises speculations (Figure 1A, Figure S1B). On the other hand, a more indirect mechanism might be considered. We know from a previous study that the β-subunit (Ca_v_β) of the L-type calcium channel (Ca_v_1.2) directly associates with the actin cytoskeleton (Stölting et al., 2015), facilitating the forward trafficking of the channel complex along actin filaments. A similar mechanism is conceivable for Ca_v_2.2 transport and translocation to the cell surface, where it accumulates, possibly via BDNF-induced F-actin assembly. This issue needs to be addressed in further studies. In fact, there are several studies discussing auxiliary subunits of N- and P/Q-type calcium channels which enhances release probability (Dolphin, 2012; Hoppa et al., 2012) and modulates axon regeneration in dorsal root ganglia (Tedeschi et al., 2016). It is tempting to speculate that BDNF/trkB signaling somehow might regulate the presence and localization of such subunits at the cell membrane, which indirectly leads to Ca_v_2.2 accumulation and spontaneous Ca^2+^ influx. In turn, the increased intracellular Ca^2+^ concentration might enhance the translocation of trkB to the cell surface (Du et al., 2000) with F-actin possibly also contributing to this process. If β2-chain laminins directly interact with actin in our experimental paradigm is not known so far.

It has been reported that local translation of β-actin mRNA in axonal growth cones of motoneurons cultured on laminin-111 is more prominent than in cells cultured on laminin-221/211 (Rathod et al., 2012). In line with this finding we observed increased β-actin immunoreactivity in axonal growth cones upon BDNF pulse on laminin-111 (Figure S5F), whereas no alterations in β-actin levels were apparent on laminin-221/211 (Figure 7D). However, on laminin-221/211 transient application of BDNF resulted in enhanced F-actin assembly (Figure 7D, Figure S5D). Furthermore, F-actin levels in axonal growth cones appear to be different *per se* between laminin-221/211 and laminin-111 (Figure S4F). On laminin-111 BDNF might stimulate local translation of β-actin in axonal growth cones of embryonic motoneurons, as shown for cortical neurons (Sasaki et al., 2010), whereas on laminin-221 BDNF rather mediates growth cone formation via F-actin assembly and stabilization. By *in situ* hybridization we detected elevated actin mRNA signal intensities upon BDNF pulse on laminin-221/211 (Figure S4A), but this does not necessarily reflect enhanced expression and translation. Furthermore, we did not distinguish between α-, β-, and γ-actin isoforms (Moradi et al., 2017), and thus the effect cannot be assigned unambiguously. It also needs to be addressed to which extent integrin signaling is involved in β2-chain laminin-mediated actin polymerization (Park and Goda, 2016) regulating growth cone formation.

In the context of motoneuron disease the presynaptic compartment with its neurotransmission machinery appears highly vulnerable to deficits in actin and microtubule dynamics. In growth cones of Smn-deficient motoneurons, β-actin mRNA levels are reduced (Rossoll et al., 2003; Jablonka et al., 2007; Rathod et al., 2012; Moradi et al., 2017). There are F-actin and tubulin deficits at motor endplates of SMA mice (Torres-Benito et al., 2011; Ackermann et al., 2013). Furthermore, this actin phenotype in the axonal compartment is accompanied by dysregulated excitability in isolated Smn-deficient motoneurons (Jablonka et al., 2007) and neurotransmission defects in neuromuscular junctions of SMA mice (Ruiz et al., 2010; Torres-Benito et al., 2011). Interestingly, SMN interacts with profilin contributing to RNA processing and neuronal transcription (Giesemann et al., 1999; Birbach, 2008; Bowerman et al., 2009). In Smn-deficient cells, hyperphosphorylation of profilin isoform 2a at serine 137/138 and reduced phospho-cofilin levels have been described changing actin dynamics and stability (Nölle et al., 2011). In line with these findings, IGF-1 downregulation results in increased Rho/ROCK levels corresponding to increased phosphorylation of profilin isoform 1 at serine 137/138 which in turn symbolizes the inactive status (Shao et al., 2008; Nölle et al., 2011; Montani and Petrinovic, 2014). In this study, we discovered that impaired BDNF/trkB signaling causes an actin- and calcium-related phenotype in growth cones of embryonic motoneurons which is also found in SMA motoneurons (Jablonka et al., 2007). Thus, it might be worthwhile investigating trkB and BDNF in SMA mouse models particularly at presynaptic structures. The importance of trkB, β2-chain laminins and voltage-gated calcium channels for neuromuscular synapse function with respect to neurotransmission and functional acetylcholine receptors is also illustrated in patients with congenital myasthenic syndromes, Lambert-Eaton myasthenic syndrome (LEMS) or myasthenia gravis (MG) (Punga and Ruegg, 2012; Shi et al., 2012).

In conclusion, we propose that foremost BDNF together with β2-chain laminin exerts specific functions in growth cone formation of embryonic motoneurons and local excitability which should be considered in the scientific discussion about pathomechanisms leading to neurotransmission defects in neuromuscular diseases.

## Author contributions

Conceived and designed the experiments: BD and SJ. Performed the experiments: BD, SB, PL, and RS. Analyzed the data: BD, SB, PL, RS, and MM. Contributed reagents/materials/analysis tools: LSB, MM, RS, and SJ. Contributed to the writing of the manuscript: SJ and BD.

### Conflict of interest statement

The authors declare that the research was conducted in the absence of any commercial or financial relationships that could be construed as a potential conflict of interest.
