# Supplementary figures and images for: BDNF/trkB Induction of Calcium Transients through Cav2.2 Calcium Channels in Motoneurons Corresponds to F-actin Assembly and Growth Cone Formation on β2-Chain Laminin (221)
Source: Front Mol Neurosci. 2017 Oct 30;10:346. doi: 10.3389/fnmol.2017.00346 (PMC5670157; doi:10.3389/fnmol.2017.00346)

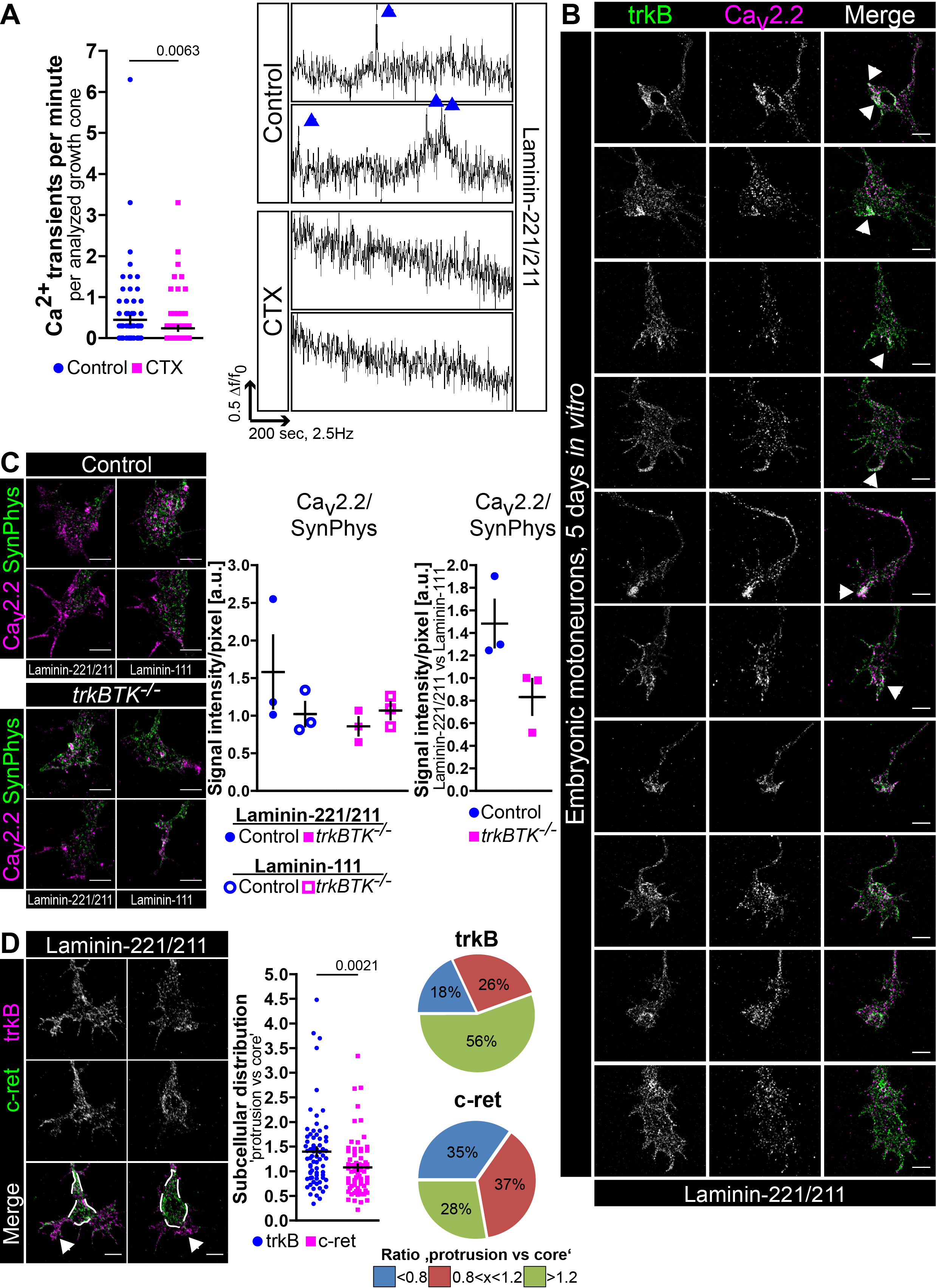

Supplement: Supplementary file 1 [file Image1.TIF]

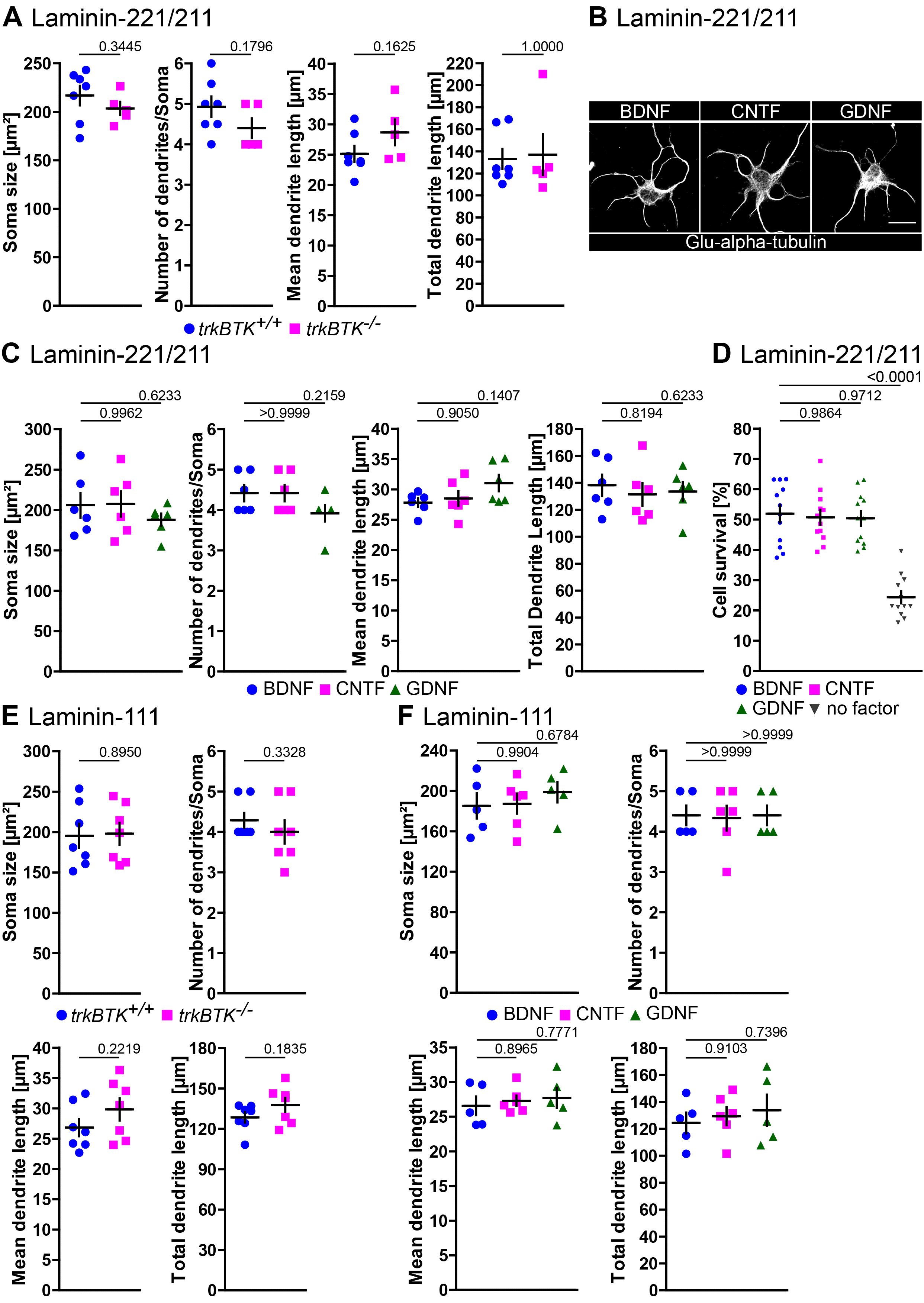

Supplement: Supplementary file 2 [file Image2.TIF]

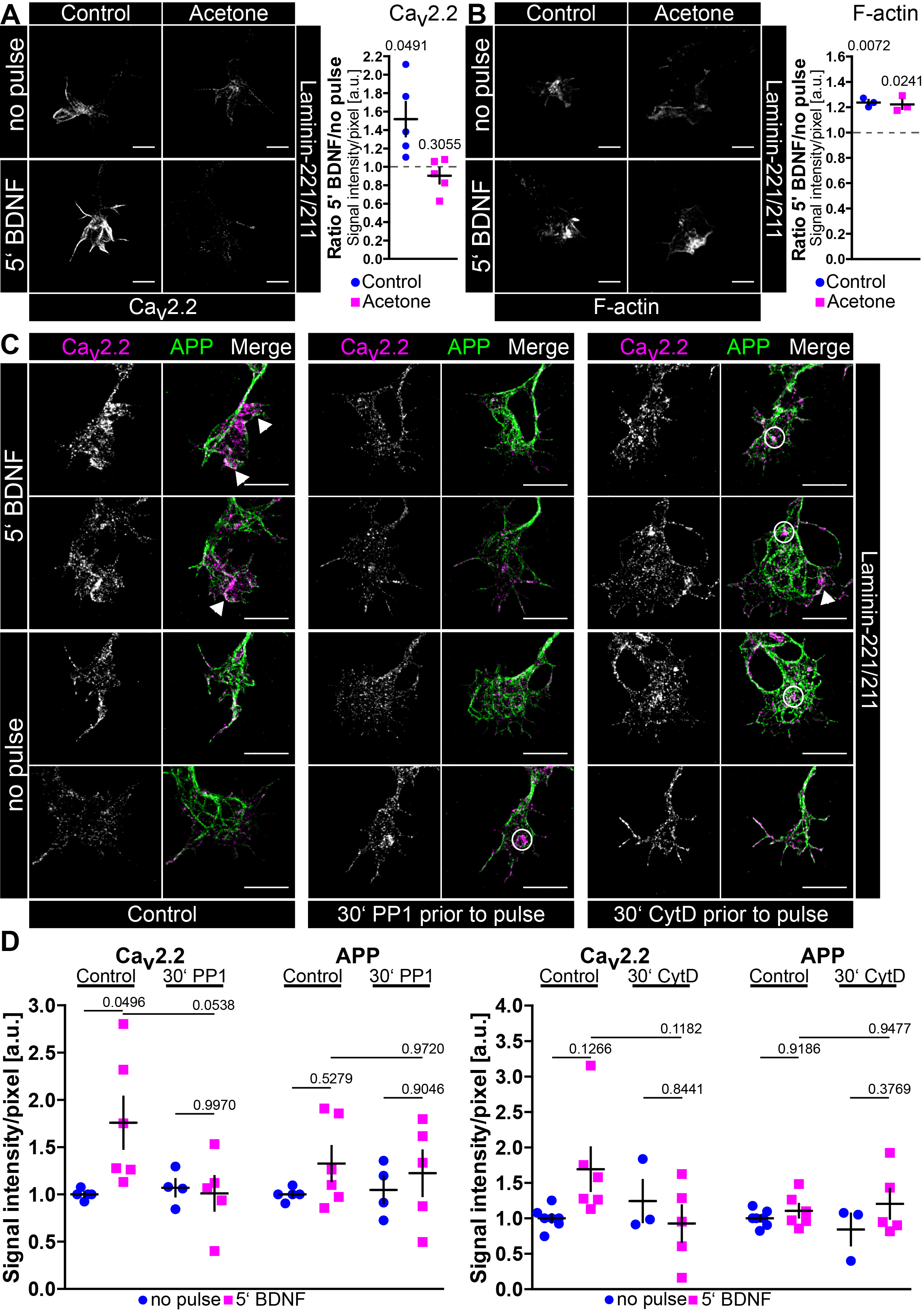

Supplement: Supplementary file 3 [file Image3.TIF]

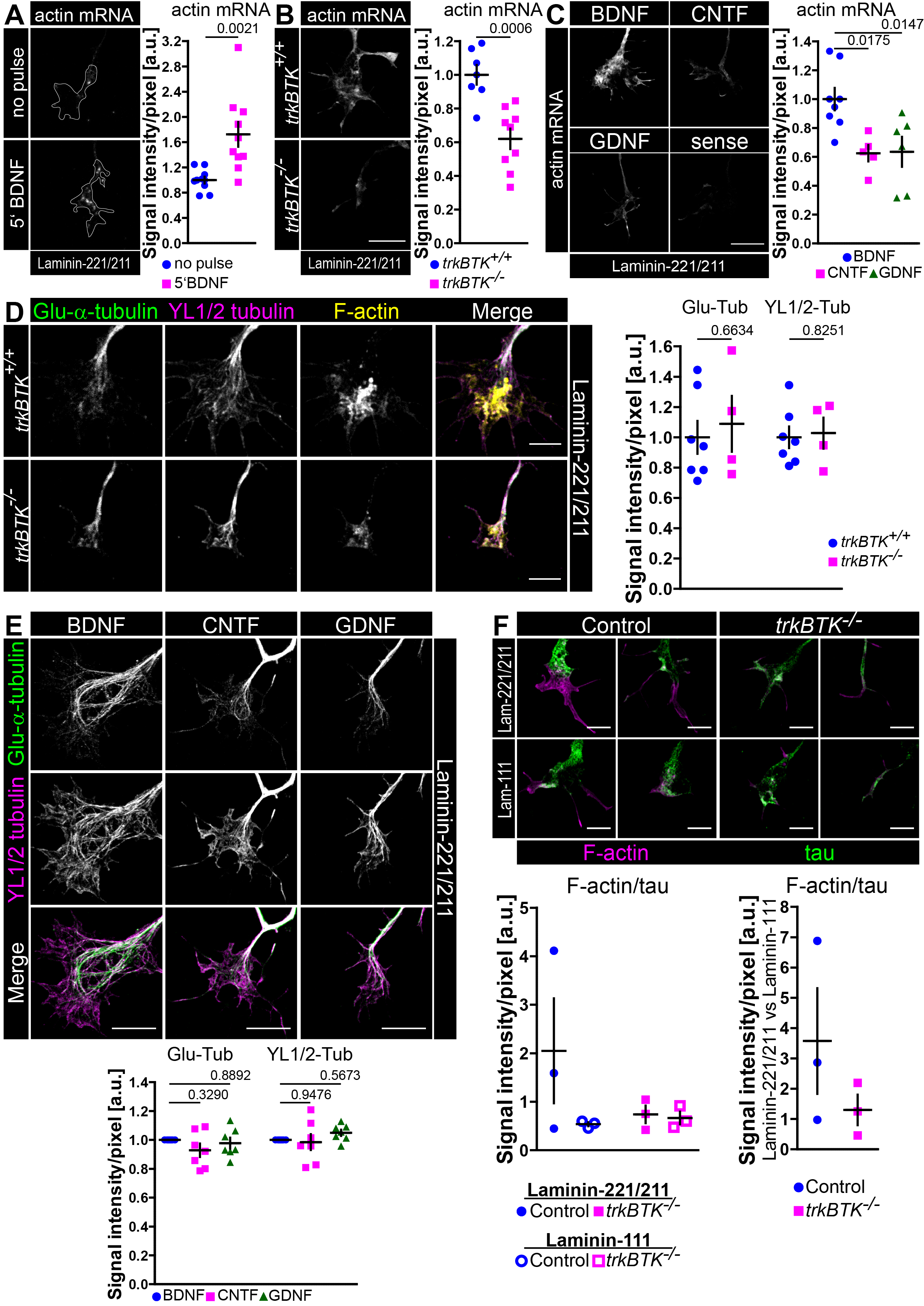

Supplement: Supplementary file 4 [file Image4.TIF]

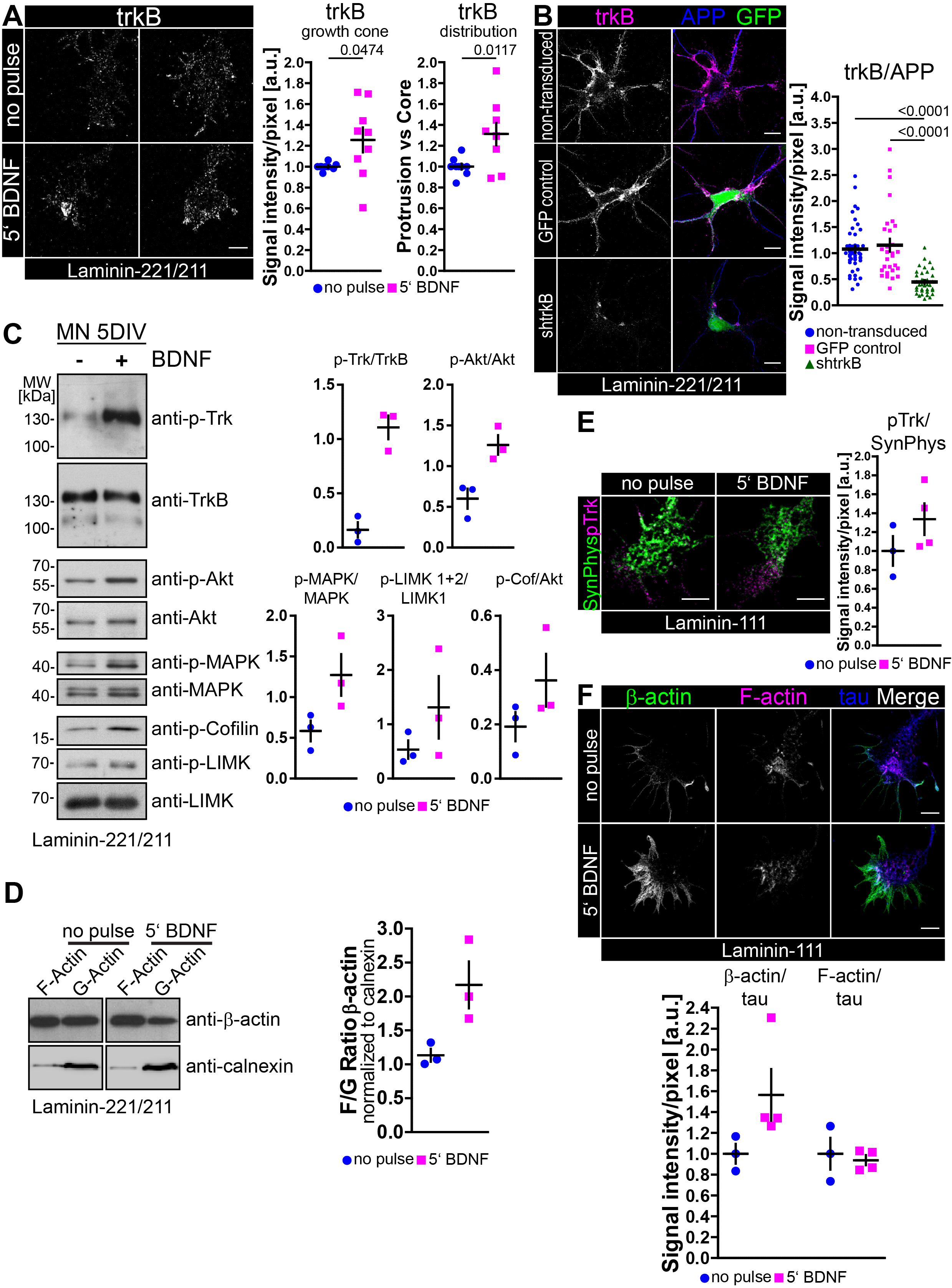

Supplement: Supplementary file 5 [file Image5.TIF]
