# Supplementary material for: BDNF/trkB Induction of Calcium Transients through Cav2.2 Calcium Channels in Motoneurons Corresponds to F-actin Assembly and Growth Cone Formation on β2-Chain Laminin (221)
Source: Front Mol Neurosci. 2017 Oct 30;10:346. doi: 10.3389/fnmol.2017.00346 (PMC5670157; doi:10.3389/fnmol.2017.00346)
Supplement: Supplementary file 8 [file DataSheet1.PDF]

Statistical analysis

Fig. 1B: $Ca_v2.2$ (*trkBTK*$^{+/+}$ 1.00 ± 0.04, Q_2 1.01, n = 6, N = 78; *trkBTK*$^{-/-}$ 0.54 ± 0.10, Q_2 0.47, n = 4, N = 66; p = 0.0015, unpaired t-test (two-tailed), t = 4.714, df = 8), $Ca_v2.2$/tau (*trkBTK*$^{+/+}$ 1.00 ± 0.08, Q_2 0.95; *trkBTK*$^{-/-}$ 0.65 ± 0.06, Q_2 0.67; p = 0.0118, unpaired t-test (two-tailed), t = 3.247, df = 8)

Fig. 1C: Ca^{2+} transients per min (Control 0.92 ± 0.13, Q_2 0.60, IQR 1.35, N = 72; *trkBTK*$^{-/-}$ 0.55 ± 0.09, Q_2 0.30, IQR 0.9, N = 64; p = 0.0337, Mann Whitney test (two-tailed), Mann Whitney U 1827)

Fig. 1D: Axon length in [µm] (*trkBTK*$^{+/+}$ 262.9 ± 11.79, Q_2 278.8, n = 7, N = 283; *trkBTK*$^{-/-}$ 316.4 ± 12.65, Q_2 314.5, n = 7, N = 298; p = 0.0093, unpaired t-test (two-tailed), t = 3.093, df = 12)

Fig. 1E: Axon length in [µm] (*trkBTK*$^{+/+}$ (A) 285.4 ± 4.26, Q_2 284.1, n = 3, N = 140; *trkBTK*$^{-/-}$ (B) 348.2 ± 4.04, Q_2 345.4, n = 3, N = 130; *trkBTK*$^{+/+}$ +CTX (C) 342.6 ± 20.20, Q_2 331.3, n = 3, N = 177; *trkBTK*$^{-/-}$ +CTX (D) 360.3 ± 7.26, Q_2 360, n = 3, N = 155; p = 0.0168 (A-B), p = 0.0273 (A-C), p = 0.0062 (A-D), one-way ANOVA with Tukey's Multiple Comparison)

Fig. 2A: $Ca_v2.2$ (BDNF (B) 1.00 ± 0.11, Q_2 1.00, n = 5, N = 130; CNTF (C) 0.65 ± 0.05, Q_2 0.65, n = 6, N = 133; GDNF (G) 0.47 ± 0.04, Q_2 0.47, n = 6, N = 145; p (B-C) = 0.0081, p (B-G) = 0.0002, one-way ANOVA with Tukey's Multiple Comparison), $Ca_v2.2$/APP (BDNF (B) 1.00 ± 0.14, Q_2 1.00, n = 5, N = 130; CNTF (C) 0.45 ± 0.05, Q_2 0.47, n = 6, N = 133; GDNF (G) 0.49 ± 0.06, Q_2 0.50, n = 6, N = 145; p (B-C) = 0.0014, p (B-G) = 0.0028, one-way ANOVA with Tukey's Multiple Comparison)

Fig. 2B: Ca^{2+} transients per min (BDNF (B) 0.89 ± 0.11, Q_2 0.6, IQR 3, N = 75; CNTF (C) 0.69 ± 0.13, Q_2 0.3, IQR 3, N = 77; GDNF (G) 0.50 ± 0.08, Q_2 0.3, IQR 2.5, N = 73; p (B-C) = 0.0306, p (B-G) = 0.0078, Kruskal-Wallis test with Dunn's multiple comparison tests)

Fig. 2C: Axon length in [µm] (BDNF (B) 356 ± 22.16, Q_2 342, n = 6, N = 275; CNTF (C) 466.6 ± 35.41, Q_2 465.6, n = 6, N = 263; GDNF (G) 454.8 ± 19.28, Q_2 440.9, n = 6, N = 200; p (B-C) = 0.0256, p (B-G) = 0.0470, one-way ANOVA with Tukey's Multiple Comparison)

Fig. 3A: $Ca_v2.2$ (no pulse 1.00 ± 0.04, Q_2 1.00, n = 9, N = 221; 5' BDNF 1.93 ± 0.24, Q_2 1.75, n = 9, N = 245; p = 0.0014, unpaired t-test (two-tailed), t = 3.839, df = 16), $Ca_v2.2$/APP (no pulse 1.00 ± 0.02, Q_2 1.00, n = 9, N = 221; 5' BDNF 1.55 ± 0.24, Q_2 1.36, n = 9, N = 245; p = 0.0370, unpaired t-test (two-tailed), t = 2.275, df = 16)

Fig. 3B: Ca^{2+} transients per min (no pulse 0.16 ± 0.08, Q_2 0, IQR 0, N = 32; 5' BDNF 0.30 ± 0.08, Q_2 0, IQR 2, N = 32; p = 0.0104, Mann Whitney test (two-tailed), Mann-Whitney U 357.5)

Fig. 3C: 5' BDNF/no pulse - $Ca_v2.2$ (*trkBTK*$^{+/+}$ 1.00 ± 0.06, Q_2 1.00, n = 7, N (no pulse) = 88, N (5' BDNF) = 102; *trkBTK*$^{-/-}$ 0.75 ± 0.07, Q_2 0.79, n = 7, N (no pulse) = 100, N (5' BDNF) = 106; p = 0.0184, unpaired t-test (two-tailed), t = 2.725, df = 12), $Ca_v2.2$/SynPhys (*trkBTK*$^{+/+}$ 1.00 ± 0.09, Q_2 1.00, IQR 0.15; *trkBTK*$^{-/-}$ 0.67 ± 0.05, Q_2 0.65, IQR 0.27; p = 0.0072, Mann Whitney test (two-tailed), Mann-Whitney U 3.000)

Fig. 4A: Growth cone size in [µm²] (Control 26.14 ± 1.13, Q_2 25.4, n = 13, N = 226; CTX 19.51 ± 1.17, Q_2 19.96, n = 17, N = 274; p = 0.0005, unpaired t-test (two-tailed), t = 3.964, df = 28), β-actin (Control 1.00 ± 0.05, Q_2 1.00; CTX 0.71 ± 0.07, Q_2 0.68; p = 0.0037, unpaired t-test (two-tailed), t = 3.170, df = 28), tau (Control 1.00 ± 0.03, Q_2 1.00; 30nM CTX 0.94 ± 0.08, Q_2 0.95; p = 0.5168, unpaired t-test (two-tailed), t = 0.6566, df = 28)

Fig. 4B: Growth cone size in [µm²] (shLUC (A) 22.02 ± 1.68, Q_2 16.89, IQR 13.07, N = 76; shActβ (B) 14.85 ± 1.18, Q_2 11.70, IQR 11.45, N = 74; Rescue (C) 23.54 ± 2.44, Q_2 17.09, IQR 13.15, N = 62; p (AB) < 0.0001, p (AC) > 0.9999, p (BC) < 0.0001, Kruskal-Wallis test with Dunn's multiple comparison tests), β-actin (shLUC (A) 1.00 ± 0.09, Q_2 0.90, IQR 0.92, N = 76; shActβ (B) 0.38 ± 0.05, Q_2 0.27, IQR 0.53, N = 74; Rescue (C) 0.98 ± 0.09, Q_2 0.96, IQR 0.82, N = 62; p (AB) < 0.0001, p (AC) > 0.9999, p (BC) < 0.0001, Kruskal-Wallis test with Dunn's multiple comparison tests), tau (shLUC (A) 1.00 ± 0.07, Q_2 0.92, IQR 0.76, N = 76; shActβ (B) 1.10 ± 0.08, Q_2 0.97, IQR 0.89, N = 74; Rescue (C) 1.07 ± 0.09, Q_2 0.90, IQR 0.73, N = 62; p (AB) > 0.9999, p (AC) > 0.9999, p (BC) > 0.9999, Kruskal-Wallis test with Dunn's multiple comparison tests)

Fig. 4C: Ca_v2.2 (shLUC (A) 1.00 ± 0.09, Q_2 0.84, IQR 0.75, N = 54; shActβ (B) 0.70 ± 0.06, Q_2 0.52, IQR 0.46, N = 63; Rescue (C) 1.04 ± 0.07, Q_2 1.05, IQR 0.90, N = 64; p (AB) = 0.0053, p (AC) > 0.9999, p (BC) = 0.0007; Kruskal-Wallis test with Dunn's multiple comparison tests), SynPhys (shLUC (A) 1.00 ± 0.08, Q_2 0.85, IQR 0.64, N = 54; shActβ (B) 1.03 ± 0.09, Q_2 0.83, IQR 0.87, N = 63; Rescue (C) 1.24 ± 0.10, Q_2 1.02, IQR 1.04, N = 64; p (AB) > 0.9999, p (AC) = 0.5022, p (BC) = 0.2002, Kruskal-Wallis test with Dunn's multiple comparison tests)

Fig. 5A: Growth cone size in [µm²] ($trkBTK^{+/+}$ 34.23 ± 2.77, Q_2 29.45, n = 20, N = 308; $trkBTK^{-/-}$ 21.22 ± 1.41, Q_2 22.81, n = 19, N = 341; p = 0.0002, unpaired t-test (two-tailed), t = 4.119, df = 37), β-actin/tau ($trkBTK^{+/+}$ 1.00 ± 0.10, Q_2 1.00, n = 8, N = 154; $trkBTK^{-/-}$ 0.55 ± 0.12, Q_2 0.45, n = 8, N = 167; p = 0.0140, unpaired t-test (two-tailed), t = 2.805, df = 14), F-actin/tau ($trkBTK^{+/+}$ 1.00 ± 0.04, Q_2 0.99, IQR 0.15, n = 19, N = 254; $trkBTK^{-/-}$ 0.76 ± 0.11, Q_2 0.64, IQR 0.44, n = 18, N = 298; p = 0.0005, Mann Whitney test (two-tailed), Mann Whitney U 60)

Fig. 5B: Growth cone size in [µm²] (BDNF (B) 31.59 ± 1.75, Q_2 31.05, n = 18, N = 385; CNTF (C) 23.01 ± 2.12, Q_2 20.73, n = 17, N = 425; GDNF (G) 21.79 ± 1.69, Q_2 18.27, n = 17, N = 435; p (B-C) = 0.0054, p (B-G) = 0.0014, one-way ANOVA with Tukey's Multiple Comparison), tau (BDNF (B) 1.00 ± 0.03, Q_2 1.00, n = 15, N = 323; CNTF (C) 1.13 ± 0.09, Q_2 1.08, n = 13, N = 336; GDNF (G) 1.05 ± 0.08, Q_2 1.07, n = 13, N = 334; p (B-C) = 0.4044, p (B-G) = 0.8411, one-way ANOVA with Tukey's Multiple Comparison), β-actin (BDNF (B) 1.00 ± 0.03, Q_2 1.00, IQR 0.03, n = 11, N = 267; CNTF (C) 0.85 ± 0.10, Q_2 0.83, IQR 0.85, n = 9, N = 274; GDNF (G) 0.73 ± 0.06, Q_2 0.70, IQR 0.73, n = 9, N = 274; p (B-C) = 0.5874, p (B-G) = 0.0163, Kruskal-Wallis test with Dunn's multiple comparisons test), F-actin (BDNF (B) 1.00 ± 0.04, Q_2 1.00, IQR 0.01, n = 14, N = 276; CNTF (C) 0.76 ± 0.10, Q_2 0.79, IQR 0.33, n = 13, N = 308; GDNF (G) 0.54 ± 0.06, Q_2 0.49, IQR 0.37, n = 13, N = 314; p (B-C) = 0.0112, p (B-G) < 0.0001, Kruskal-Wallis test with Dunn's multiple comparisons test)

Fig. 6A: Axon length in [µm] ($trkBTK^{+/+}$ 468.8 ± 39.31, Q_2 454.9, n = 9, N = 615; $trkBTK^{-/-}$ 357.2 ± 29.33, Q_2 363, n = 8, N = 579; p = 0.0417, unpaired t-test (two-tailed), t = 2.227, df = 15)

Fig. 6C: Growth cone size in [µm²] (*trkBTK*$^{+/+}$ 27.51 ± 4.58, Q_2 25.06, n = 7, N = 113; *trkBTK*$^{-/-}$ 16.55 ± 1.26, Q_2 17.12, n = 11, N = 178; p = 0.0132, unpaired t-test (two-tailed), t = 2.788, df = 16), β-actin (*trkBTK*$^{+/+}$ 1.00 ± 0.07, Q_2 0.99, n = 7, N = 113; *trkBTK*$^{-/-}$ 1.11 ± 0.07, Q_2 1.15, n = 11, N = 178; p = 0.2696, unpaired t-test (two-tailed), t = 1.144, df = 16), F-actin (*trkBTK*$^{+/+}$ 1.00 ± 0.09, Q_2 1.00, n = 7, N = 113; *trkBTK*$^{-/-}$ 0.81 ± 0.06, Q_2 0.80, n = 11, N = 178; p = 0.0819, unpaired t-test (two-tailed), t = 1.856, df = 16), tau (*trkBTK*$^{+/+}$ 1.00 ± 0.06, Q_2 1.00, n = 7, N = 113; *trkBTK*$^{-/-}$ 1.40 ± 0.19, Q_2 1.25, n = 11, N = 178; p = 0.1287, unpaired t-test (two-tailed), t = 1.602, df = 16)

Fig. 6D: Axon length in [µm] (BDNF (B) 425.3 ± 14.93, Q_2 423.3, n = 5, N = 256; CNTF (C) 439.0 ± 18.37, Q_2 439.9, n = 6, N = 258; GDNF (G) 388.6 ± 24.47, Q_2 412.1, n = 5, N = 273; p (B-C) = 0.8710, p (B-G) = 0.4295, one-way ANOVA with Tukey's Multiple Comparison)

Fig. 6F: Growth cone size in [µm²] (BDNF (B) 32.83 ± 4.93, Q_2 29.04, n = 6, N = 165; CNTF (C) 33.01 ± 4.41, Q_2 30.96, n = 6, N = 175; GDNF (G) 37.85 ± 4.72, Q_2 38.52, n = 6, N = 172; p (B-C) = 0.9996, p (B-G) = 0.7352, one-way ANOVA with Tukey's Multiple Comparison), F-actin (BDNF (B) 1.00 ± 0.02, Q_2 1.00, IQR 0.04, n = 6, N = 165; CNTF (C) 1.14 ± 0.22, Q_2 0.95, IQR 0.97, n = 6, N = 175; GDNF (G) 0.99 ± 0.28, Q_2 0.79, IQR 1.02, n = 6, N = 172; p (B-C) > 0.9999, p (B-G) > 0.9999, Kruskal-Wallis test with Dunn's multiple comparisons test), tau (BDNF (B) 1.00 ± 0.03, Q_2 1.00, IQR 0.05, n = 6, N = 165; CNTF (C) 0.92 ± 0.08, Q_2 0.93, IQR 0.34, n = 6, N = 175; GDNF (G) 0.82 ± 0.11, Q_2 0.82, IQR 0.43, n = 6, N = 172; p (B-C) > 0.9999, p (B-G) = 0.3449, Kruskal-Wallis test with Dunn's multiple comparisons test), F-actin/tau (BDNF (B) 1.00 ± 0.08, Q_2 1.00, IQR 0.15, n = 6, N = 165; CNTF (C) 1.25 ± 0.16, Q_2 1.05, IQR 0.52, n = 6, N = 175; GDNF (G) 1.12 ± 0.24, Q_2 1.14, IQR 1.13, n = 6, N = 172; p (B-C) = 0.2459, p (B-G) = 0.6953, Kruskal-Wallis test with Dunn's multiple comparisons test)

Fig. 7A: pTrk/SynPhys (no pulse 1.00 ± 0.08, Q_2 1.00, n = 10, N = 136; 5' BDNF 1.72 ± 0.25, Q_2 1.63, n = 10, N = 137; p = 0.0154, unpaired t-test (two-tailed), t = 2.677, df = 18)

Fig. 7B: pCof/Cofilin (no pulse 1.00 ± 0.03, Q_2 1.00, n = 11, N = 174; 5' BDNF 1.73 ± 0.17, Q_2 1.69, n = 11, N = 176; p = 0.0003, unpaired t-test (two-tailed), t = 4.303, df = 20), pCof/SynPhys (no pulse 1.00 ± 0.04, Q_2 1.00, n = 11, N = 174; 5' BDNF 1.68 ± 0.15, Q_2 1.70, n = 11, N = 176; p = 0.0003, unpaired t-test (two-tailed), t = 4.424, df = 20)

Fig. 7C: pProf/SynPhys (no pulse 1.00 ± 0.04, Q_2 1.00, IQR 0.09, n = 13, N = 200; 5' BDNF 1.59 ± 0.12, Q_2 1.67, IQR 0.57, n = 14, N = 226; p = 0.0012, Mann Whitney test (two-tailed), Mann-Whitney U 24)

Fig. 7D: β-actin (no pulse 1.00 ± 0.07, Q_2 1.00, n = 7, N = 194; 5' BDNF 1.00 ± 0.09, Q_2 0.98, n = 9, N = 236; p = 0.9861, unpaired t-test (two-tailed), t = 0.0178, df = 14), F-actin (no pulse 1.00 ± 0.06, Q_2 1.00, n = 7, N = 194; 5' BDNF 1.49 ± 0.13, Q_2 1.48, n = 9, N = 236; p = 0.0088, unpaired t-test (two-tailed), t = 3.040, df = 14), tau (no pulse 1.00 ± 0.05, Q_2 1.00, n = 7, N = 194; 5' BDNF 1.06 ± 0.05, Q_2 1.08, n = 9, N = 236; p = 0.4391, unpaired t-test (two-tailed), t = 0.7965, df = 14)

Fig. 7F: Velocity in µm/sec (no pulse 0.25 ± 0.02, Q_2 0.27, IQR 0.20, N = 49; 5' BDNF 0.18 ± 0.02, Q_2 0.14, IQR 0.16, N = 44; p = 0.0044, Mann Whitney test (two-tailed), Mann-Whitney U 707)

Fig. S1A: Ca^{2+} transients per min (Control 0.45 ± 0.09, Q_2 0.30, IQR 0.6, N = 85; CTX 0.24 ± 0.06, Q_2 0.00, IQR 0.3, N = 86; p = 0.0063, Mann Whitney test (two-tailed), Mann Whitney U 2873)

Fig. S1C: Ca_v2.2/SynPhys (Control_Laminin-221/211 1.58 ± 0.49, Q_2 1.18, n = 3, N = 32; Control_Laminin-111 1.02 ± 0.16, Q_2 0.91, n = 3, N = 38; *trkBTK*$^{-/-}$_Laminin-221/211 0.86 ± 0.12, Q_2 0.86, n = 3, N = 38; *trkBTK*$^{-/-}$_Laminin-111 1.07 ± 0.12, Q_2 1.09, n = 3, N = 36), Ca_v2.2/SynPhys – Laminin-221/211 vs Laminin-111 (Control 1.48 ± 0.21, Q_2 1.30, n = 3; *trkBTK*$^{-/-}$ 0.83 ± 0.16, Q_2 0.98, n = 3)

Fig. S1D: Protrusion vs core (trkB 1.40 ± 0.09, Q_2 1.28, IQR 0.81; c-ret 1.08 ± 0.07, Q_2 1.00, IQR 0.61; N = 72, p = 0.0021, Mann Whitney test (two-tailed), Mann Whitney U 1820)

Fig. S2A: Soma size in [µm²] (*trkBTK*$^{+/+}$ 216.9 ± 10.13, Q_2 226.4, n = 7, N = 124; *trkBTK*$^{-/-}$ 203.6 ± 6.815, Q_2 201.6, n = 5, N = 72; p = 0.3445, unpaired t-test (two tailed), t = 0.9921, df = 10), number of dendrites per soma (*trkBTK*$^{+/+}$ 4.93 ± 0.25, Q_2 5, n = 7, N = 124; *trkBTK*$^{-/-}$ 4.40 ± 0.24, Q_2 4, n = 5, N = 72; p = 0.1796, unpaired t-test (two tailed), t = 1.443, df = 10), mean dendrite length in [µm] (*trkBTK*$^{+/+}$ 25.13 ± 1.31, Q_2 23.9, n = 7, N = 640; *trkBTK*$^{-/-}$ 28.67 ± 2.11, Q_2 28.29, n = 5, N = 328; p = 0.1625, unpaired t-test (two tailed), t = 1.508, df = 10), total dendrite length in [µm] (*trkBTK*$^{+/+}$ 133 ± 9.14, Q_2 124.1, IQR 48.1, n = 7, N = 124; *trkBTK*$^{-/-}$ 137.1 ± 18.52, Q_2 123.1, IQR 54.4, n = 5, N = 72; p = 1.0000, Mann Whitney (two tailed), Mann Whitney U 17)

Fig. S2C: Soma size in [µm²] (BDNF (B) 205.8 ± 13.37, Q_2 195.3, n = 6, N = 106; CNTF (C) 207.4 ± 15.69, Q_2 207.2, n = 6, N = 106; GDNF (G) 187.9 ± 7.59, Q_2 193.9, n = 6, N = 96; p (B-C) = 0.9962, p (B-G) = 0.6233, one-way ANOVA with Tukey's Multiple Comparison), number of dendrites per soma (BDNF (B) 4.42 ± 0.20, Q_2 4.25, n = 6, N = 106; CNTF (C) 4.42 ± 0.20, Q_2 4.25, n = 6, N = 106; GDNF (G) 3.92 ± 0.20, Q_2 4, n = 6, N = 96; p (B-C) > 0.9999, p (B-G) = 0.2159, one-way ANOVA with Tukey's Multiple Comparison), mean dendrite length in [µm] (BDNF (B) 27.81 ± 0.70, Q_2 28.28, n = 6, N = 488; CNTF (C) 28.49 ± 1.21, Q_2 27.8, n = 6, N = 457; GDNF (G) 31.03 ± 1.36, Q_2 30, n = 6, N = 391; p (B-C) = 0.9050, p (B-G) = 0.1407, one-way ANOVA with Tukey's Multiple Comparison), total dendrite length in [µm] (BDNF (B) 138.2 ± 7.92, Q_2 134.5, n = 6, N = 106; CNTF (C) 131.5 ± 8.41, Q_2 127.1, n = 6, N = 106; GDNF (G) 133.6 ± 7.00, Q_2 137.4, n = 6, N = 96; p (B-C) = 0.8194, p (B-G) = 0.6233, one-way ANOVA with Tukey's Multiple Comparison)

Fig. S2D: Cell survival in % (BDNF (B) 51.91 ± 2.87, Q_2 53.19, n = 12; CNTF (C) 50.74 ± 2.46, Q_2 49.89, n = 12; GDNF (G) 50.38 ± 2.44, Q_2 50.37, n = 12; no factor (NF) 24.35 ± 2.00, Q_2 21.48, n = 12; p (B-C) = 0.9864, p (B-G) = 0.9712, p (B-NF) < 0.0001, one-way ANOVA with Tukey's Multiple Comparison)

Fig. S2E: Soma size in [µm²] (*trkBTK*$^{+/+}$ 195.2 ± 14.97, Q_2 181, n = 7, N = 143; *trkBTK*$^{-/-}$ 198.0 ± 13.46, Q_2 199.1, n = 7, N = 124; p = 0.8950, unpaired t-test (two tailed), t = 0.1348, df = 12), number of dendrites per soma (*trkBTK*$^{+/+}$ 4.29 ± 0.18, Q_2 4, IQR 1, n = 7, N = 143; *trkBTK*$^{-/-}$ 4.00 ± 0.29, Q_2 4, IQR 1.5, n = 7, N = 124; p = 0.3328, Mann Whitney (two tailed), Mann Whitney U 17), mean dendrite length in [µm] (*trkBTK*$^{+/+}$ 26.82 ± 1.42, Q_2 26.09, n = 7, N = 661; *trkBTK*$^{-/-}$ 29.82 ± 1.84, Q_2 30.53, n = 7, N = 560; p = 0.2219, unpaired t-test (two tailed), t = 1.288, df = 12), total dendrite length in [µm] (*trkBTK*$^{+/+}$ 128.5 ± 3.91, Q_2 133.1, n = 7, N = 143; *trkBTK*$^{-/-}$ 137.8 ± 5.26, Q_2 143, n = 7, N = 124; p = 0.1835, unpaired t-test (two-tailed), t = 1.411, df = 12)

Fig. S2F: Soma size in [µm²] (BDNF (B) 185.3 ± 12.64, Q_2 181.4, n = 5, N = 99; CNTF (C) 187.3 ± 9.87, Q_2 195.1, n = 6, N = 124; GDNF (G) 198.8 ± 10.12, Q_2 201.1, n = 5, N = 124; p (B-C) = 0.9904, p (B-G) = 0.6784, one-way ANOVA with Tukey's Multiple Comparison), number of dendrites per soma (BDNF (B) 4.40 ± 0.24, Q_2 4.00, IQR 1.00, n = 5, N = 99; CNTF (C) 4.33 ± 0.31, Q_2 4.50, IQR 1.25, n = 6, N = 124; GDNF (G) 4.40 ± 0.24, Q_2 4.00, IQR 1.00, n = 5, N = 124; p (B-C) > 0.9999, p (B-G) > 0.9999, Kruskal-Wallis test with Dunn's multiple comparisons test), mean dendrite length in [µm] (BDNF (B) 26.58 ± 1.34, Q_2 25.61, n = 5, N = 447; CNTF (C) 27.30 ± 0.75, Q_2 26.9, n = 6, N = 583; GDNF (G) 27.74 ± 1.43, Q_2 27.04, n = 5, N = 517; p (B-C) = 0.8965, p (B-G) = 0.7771, one-way ANOVA with Tukey's Multiple Comparison), total dendrite length in [µm] (BDNF (B) 124.4 ± 7.66, Q_2 127.7, n = 5, N = 99; CNTF (C) 129.4 ± 6.74, Q_2 130.3, n = 6, N = 124; GDNF (G) 133.9 ± 11.54, Q_2 125.5, n = 5, N = 124; p (B-C) = 0.9103, p (B-G) = 0.7396, one-way ANOVA with Tukey's Multiple Comparison)

Fig. S3A: $Ca_v2.2$ – 5' BDNF vs no pulse (Control 1.52 ± 0.19, Q_2 1.38, n = 5, N (no pulse) = 129, N (5' BDNF) = 140; Acetone 0.90 ± 0.08, Q_2 0.92, n = 5, N (no pulse) = 140, N (5' BDNF) = 145; p (Control) = 0.0491, t = 2.793, df = 4; p (Acetone) = 0.3055, t = 1.174, df = 4; one sample t-test with theoretical mean set as 1)

Fig. S3B: F-actin – 5' BDNF vs no pulse (Control 1.24 ± 0.02, Q_2 1.24, n = 3, N (no pulse) = 118, N (5' BDNF) = 116; Acetone 1.22 ± 0.04, Q_2 1.20, n = 3, N (no pulse) = 119, N (5' BDNF) = 91; p (Control) = 0.0072, t = 11.73, df = 2; p (Acetone) = 0.0241, t = 6.329, df = 2; one sample t-test with theoretical mean set as 1)

Fig. S3D: PP1 – $Ca_v2.2$ (Control_no pulse (A) 1.00 ± 0.02, Q_2 1.00, n = 5, N = 136; Control_5' BDNF (B) 1.76 ± 0.28, Q_2 1.51, n = 6, N = 169; PP1_no pulse (C) 1.07 ± 0.09, Q_2 1.07, n = 4, N = 116; PP1_5' BDNF (D) 1.01 ± 0.18, Q_2 1.07, n = 5, N = 130; p (AB) = 0.0496, p (BD) = 0.0538, p (CD) = 0.9970, one-way ANOVA with Tukey's Multiple Comparison), PP1 – APP (Control_no pulse (A) 1.00 ± 0.03, Q_2 1, n = 5, N = 136; Control_5' BDNF (B) 1.33 ± 0.18, Q_2 1.18, n = 6, N = 169; PP1_no pulse (C) 1.05 ± 0.14, Q_2 1.06, n = 4, N = 116; PP1_5' BDNF (D) 1.22 ± 0.24, Q_2 1.34, n = 5, N = 130; p (AB) = 0.5279, p (BD) = 0.9720, p (CD) = 0.9046, one-way ANOVA with Tukey's Multiple Comparison), CytD – $Ca_v2.2$ (Control_no pulse (A) 1.00 ± 0.06, Q_2 1.00, n = 7, N = 166; Control_5' BDNF (B) 1.69 ± 0.31, Q_2 1.43, n = 6, N = 151; CytD_no pulse (C) 1.24 ± 0.30, Q_2 0.98, n = 3, N = 85; CytD_5' BDNF (D) 0.93 ± 0.26, Q_2 0.95, n = 5, N = 114; p (AB) = 0.1266, p (BD) = 0.1182, p (CD) = 0.8441, one-way ANOVA with Tukey's Multiple Comparison), CytD – APP (Control_no pulse (A) 1.00 ± 0.04, Q_2 1.00, n = 7, N = 166; Control_5' BDNF (B) 1.11 ± 0.09, Q_2 1.04, n = 6, N = 151; CytD_no pulse (C) 0.84 ± 0.22, Q_2 1.05, n = 3, N = 85; CytD_5' BDNF (D) 1.20 ± 0.21, Q_2 0.95, n = 5, N = 114; p (AB) = 0.9186, p (BD) = 0.9477, p (CD) = 0.3769, one-way ANOVA with Tukey's Multiple Comparison; the BDNF pulse *per se* resulted in increased $Ca_v2.2$ immunoreactivity under control conditions, unpaired t-test (two-tailed), p = 0.0353, t = 2.400, df = 11)

Fig. S4A: actin mRNA (no pulse 1.00 ± 0.05, Q_2 1.00, n = 10, N = 225; 5' BDNF 1.72 ± 0.19, Q_2 1.56, n = 10, N = 233; p = 0.0021, unpaired t-test (two-tailed), t = 3.583, df = 18)

Fig. S4B: actin mRNA (*trkBTK*$^{+/+}$ 1.00 ± 0.06, Q_2 1.00, n = 7, N = 190; *trkBTK*$^{-/-}$ 0.62 ± 0.06, Q_2 0.69, n = 9, N = 229; p = 0.0006, unpaired t-test (two-tailed), t = 4.429, df = 14)

Fig. S4C: actin mRNA (BDNF (B) 1.00 ± 0.08, Q_2 0.97, n = 8, N = 186; CNTF (C) 0.62 ± 0.06, Q_2 0.63, n = 5, N = 148; GDNF (G) 0.63 ± 0.10, Q_2 0.72, n = 6, N = 211; p (B-C) = 0.0175, p (B-G) = 0.0147, one-way ANOVA with Tukey's Multiple Comparison)

Fig. S4D: Glu-Tub (*trkBTK*$^{+/+}$ 1.00 ± 0.11, Q_2 0.94, n = 7, N = 91; *trkBTK*$^{-/-}$ 1.09 ± 0.18, Q_2 1.01, n = 4, N = 66; p = 0.6634, unpaired t-test (two-tailed), t = 0.4500, df = 9), YL1/2-Tub (*trkBTK*$^{+/+}$ 1.00 ± 0.07, Q_2 0.97, n = 7, N = 91; *trkBTK*$^{-/-}$ 1.03 ± 0.10, Q_2 1.06, n = 4, N = 66; p = 0.8251, unpaired t-test (two-tailed), t = 0.2275, df = 9)

Fig. S4E: Glu-Tub (BDNF (B) 1.00, Q_2 1.00, n = 7, N = 198; CNTF (C) 0.93 ± 0.05, Q_2 0.92, n = 7, N = 186; GDNF (G) 0.98 ± 0.04, Q_2 0.93, n = 7, N = 180; p (B-C) = 0.3290, p (B-G) = 0.8892, one-way ANOVA with Tukey's Multiple Comparison), YL1/2-Tub (BDNF (B) 1.00, Q_2 1.00, n = 7, N = 198; CNTF (C) 0.98 ± 0.05, Q_2 0.96, n = 7, N = 186; GDNF (G) 1.05 ± 0.02, Q_2 1.05, n = 7, N = 180; p (B-C) = 0.9476, p (B-G) = 0.5673, one-way ANOVA with Tukey's Multiple Comparison)

Fig. S4F: F-actin/tau (Control_Laminin-221/211 2.05 ± 1.08, Q_2 1.59, n = 3, N = 20; Control_Laminin-111 0.54 ± 0.04, Q_2 0.55, n = 3, N = 25; *trkBTK*$^{-/-}$_Laminin-221/211 0.74 ± 0.18, Q_2 0.75, n = 3, N = 26; *trkBTK*$^{-/-}$_Laminin-111 0.66 ± 0.13, Q_2 0.60, n = 3, N = 25), F-actin/tau – Laminin-221/211 vs Laminin-111 (Control 3.57 ± 1.74, Q_2 2.87, n = 3; *trkBTK*$^{-/-}$ 1.30 ± 0.50, Q_2 1.25, n = 3)

Fig. S5A: trkB – immunoreactivity (no pulse 1.00 ± 0.01, Q_2 1.00, n = 9, N = 165; 5' BDNF 1.26 ± 0.12, Q_2 1.33, n = 9, N = 139; p = 0.0474, unpaired t-test (two-tailed), t = 2.148, df = 16), trkB – protrusion vs core (no pulse 1.00 ± 0.03, Q_2 1.00, n = 9, N = 165; 5' BDNF 1.31 ± 0.11, Q_2 1.29, n = 9, N = 139; p = 0.0117, unpaired t-test (two-tailed), t = 2.843, df = 16)

Fig. S5B: trkB/APP (non-transduced (A) 1.08 ± 0.06, Q_2 1.00, IQR 0.28, N = 49; GFP control (B) 1.15 ± 0.12, Q_2 1.00, IQR 0.82, N = 29; shtrkB (C) 0.45 ± 0.04, Q_2 0.40, IQR 0.31, N = 37; p (AB) > 0.9999, p (AC) < 0.0001, p (BC) < 0.0001, Kruskal-Wallis test with Dunn's multiple comparison tests)

Fig. S5C: pTrk/TrkB (no pulse 0.16 ± 0.07, Q_2 0.15, n = 3; 5' BDNF 1.11 ± 0.11, Q_2 1.21, n = 3), pAKT/AKT (no pulse 0.60 ± 0.12, Q_2 0.71, n = 3; 5' BDNF 1.26 ± 0.12, Q_2 1.20, n = 3), pMAPK/MAPK (no pulse 0.58 ± 0.13, Q_2 0.63, n = 3; 5' BDNF 1.27 ± 0.25, Q_2 1.17, n = 3), pLIMK1+2/LIMK1 (no pulse 0.53 ± 0.17, Q_2 0.42, n = 3; 5' BDNF 1.31 ± 0.58, Q_2 1.11, n = 3), pCof/AKT (no pulse 0.19 ± 0.05, Q_2 0.22, n = 3; 5' BDNF 0.36 ± 0.10, Q_2 0.27, n = 3)

Fig. S5D: F/G Ratio β-actin (no pulse 1.13 ± 0.10, Q_2 1.07, n = 3; 5' BDNF 2.17 ± 0.35, Q_2 2.00, n = 3)

Fig. S5E: pTrk/SynPhys (no pulse 1.00 ± 0.16, Q_2 1.00, n = 3, N = 56; 5' BDNF 1.34 ± 0.17, Q_2 1.27, n = 4, N = 68)

Fig. S5F: β-actin/tau (no pulse 1.00 ± 0.10, Q_2 1.00, n = 3, N = 48; 5' BDNF 1.56 ± 0.25, Q_2 1.34, n = 4, N = 68), F-actin/tau (no pulse 1.00 ± 0.15, Q_2 1.00, n = 3, N = 48; 5' BDNF 0.94 ± 0.05, Q_2 0.94, n = 4, N = 68)
