# Supplementary material for: BDNF/trkB Induction of Calcium Transients through Cav2.2 Calcium Channels in Motoneurons Corresponds to F-actin Assembly and Growth Cone Formation on β2-Chain Laminin (221)
Source: Front Mol Neurosci. 2017 Oct 30;10:346. doi: 10.3389/fnmol.2017.00346 (PMC5670157; doi:10.3389/fnmol.2017.00346)
Supplement: Supplementary file 9 [file DataSheet2.docx]

**Supplementary Figures**

**Figure S1: Distribution of trkB, Ca_v_2.2 and c-ret in axonal growth cones of embryonic motoneurons cultured on laminin-221/211.** (A) Treatment with 30nM ω-conotoxin (CTX) significantly (p=0.0063) reduced the frequency of spontaneous Ca^2+^ transients (highlighted by blue arrowheads) in control motoneurons cultured on laminin-221. Representative traces are shown in the right panel. (B) Representative images of growth cones of embryonic motoneurons cultured on laminin-221/211 for 5 days *in vitro* in the presence of BDNF and CNTF and stained against trkB (green) and Ca_v_2.2 (magenta) (scale bar: 5µm). TrkB and Ca_v_2.2 signals accumulated in protrusions of axonal growth cones in close proximity as highlighted by white arrowheads. (C) Control growth cones revealed higher levels of Ca_v_2.2 immunoreactivity (magenta) in relation to synaptophysin (SynPhys, green) on laminin-221/211 than on laminin-111, whereas *trkBTK^-/-^* growth cones appeared to display equal Ca_v_2.2/SynPhys intensities independently of the used laminin isoform (scale bar: 5µm). On laminin-221/211 a difference in Ca_v_2.2/SynPhys was detectable between both genotypes, whereas on laminin-111 immunofluorescences were comparable. (D) Wild type motoneurons were cultured on laminin-221/211 for 5 days *in vitro* in the presence of BDNF, CNTF and GDNF and stained against c-ret (green) and trkB (magenta) (scale bar: 5µm). TrkB was mainly located at protrusions, as indicated by white arrowheads, whereas c-ret immunoreactivity was concentrated rather within central regions, as highlighted by dotted white polygons. The ratio between protrusion and core signal intensity was significantly higher in the case of trkB than of c-ret (trkB 1.40 ± 0.09, Q_2_ 1.28, IQR 0.81; c-ret 1.08 ± 0.07, Q_2_ 1.00, IQR 0.61; N=72, p=0.0021). The relative percentage of growth cones displaying a protrusion-oriented location, i.e., values greater than 1.2 (green), was much greater in the case of trkB than of c-ret (56% vs 28%). *Vice versa*, the number of growth cones showing a core-oriented distribution, i.e., values lower than 0.8 (blue), was more increased in the case of c-ret than of trkB (35% vs 18%). The relative percentage of axon terminals exhibiting a balanced dispersion between both compartments, i.e., values between 0.8 and 1.2 (red), was 26% in the case of trkB and 37% in the case of c-ret. (D) Modified from @Dombert, B. (2014). Molecular mechanisms underlying defective axonal growth and presynaptic differentiation in spinal muscular atrophy. Universitaet Wuerzburg.

**Figure S2: Dendrite complexity is not altered in *trkBTK^-/-^* and BDNF-deprived motoneurons.** (A) *TrkBTK^+/+^* and *trkBTK^-/-^* motoneurons were cultured on laminin-221/211 for 7 days *in vitro* in the presence of BDNF and CNTF. There were no obvious differences in soma size, number of dendrites per soma, mean dendrite length and total dendrite length between both genotypes. (B) Representative images of BDNF-, CNTF- and GDNF-treated motoneurons cultured on laminin-221/211 for 7 days *in vitro* and stained against Glu-alpha-tubulin (scale bar: 10µm). (C) Soma size and dendrite complexity, i.e., number of dendrites per soma, mean dendrite length and total dendrite length, were statistically comparable in each condition. (D) BDNF, CNTF and GDNF equally promoted cell survival on laminin-221/211. Without neurotrophic support the survival rate of motoneurons was significantly reduced. (E) TrkB mutant motoneurons cultured on laminin-111 for 7 days *in vitro* did not develop any morphological alterations with respect to soma size and dendrite complexity in comparison to wild type controls. (F) Similar results were obtained with wild type motoneurons cultured on laminin-111 for 7 days *in vitro* in the presence of BDNF, CNTF or GDNF.

**Figure S3: BDNF-induced Ca_v_2.2 clustering on laminin-221/211 in axonal growth cones is reduced by pharmacological manipulation of Src kinases and actin cytoskeleton.** (A) Treatment of fixed motoneurons with acetone during the staining procedure negated the BDNF-induced effect on Ca_v_2.2 accumulation in axonal growth cones (5’ BDNF/no pulse Control 1.52 ± 0.19, Q_2_ 1.38, n=5, N (no pulse) = 129, N (5’ BDNF) = 140; Acetone 0.90 ± 0.08, Q_2_ 0.92, n=5, N (no pulse) = 140, N (5’ BDNF) = 145; p (Control) = 0.0491, p (Acetone) = 0.3055; tested against hypothetical value of 1). (B) The BDNF-mediated effect on F-actin assembly was not affected by acetone treatment. (C) Representative images of axonal growth cones of wild type motoneurons cultured on laminin-221/211 for 5 days *in vitro* with and without BDNF pulse and with and without pre-treatment with Src kinase inhibitor cocktail PP1 or cytochalasin D (CytD), respectively (scale bar: 5µm). Cells were stained against Ca_v_2.2 (magenta) and APP (green). Under control conditions clustering of Ca_v_2.2 channels was enhanced upon BDNF pulse, particularly at growth cone protrusions (indicated by white arrowheads). This BDNF-mediated effect was impaired by pre-treatment of cells with either PP1 or CytD for 30 minutes. There was no increase in Ca_v_2.2 signals in comparison to the corresponding non-pulsed growth cones. Few Ca_v_2.2 clusters were rather detected in core regions of axon terminals than in growth cone tips, as highlighted by white circles. APP levels were comparable in each analyzed condition. (D) Corresponding statistical analysis of Ca_v_2.2 and APP intensities with and without BDNF, PP1 or CytD.

**Figure S4: Impaired BDNF/trkB signaling lowers actin mRNA in axonal growth cones of motoneurons cultured on laminin-221/211, whereas glutamylated and tyrosinated tubulin levels appear not affected.** (A) Upon BDNF pulse actin mRNA immunoreactivity was significantly enhanced in growth cones of embryonic motoneurons cultured on laminin-221/211 for 5 days *in vitro* (no pulse 1.00 ± 0.05, Q_2_ 1.00, n=10, N=225; 5’ BDNF 1.72 ± 0.19, Q_2_ 1.56, n=10, N=233; p=0.0021) (scale bar: 5µm). (B) Images of axonal growth cones of *trkBTK^+/+^* and *trkBTK^-/-^* motoneurons cultured on laminin-221/211 for 5 days *in vitro* and stained against actin mRNA by *in situ* hybridization (scale bar: 5µm). In *trkBTK^-/-^* growth cones actin mRNA immunoreactivity was significantly reduced in comparison to wild type controls (*trkBTK^+/+^* 1.00 ± 0.06, Q_2_ 1.00, n=7, N=190; *trkBTK^-/-^* 0.62 ± 0.06, Q_2_ 0.69, n=9, N=229; p=0.0006). (C) Similar results were obtained with motoneurons cultured on laminin-221/211 with BDNF, CNTF, or GDNF (scale bar: 5µm). In the presence of BDNF higher actin mRNA signals (1.00 ± 0.08, Q_2_ 0.97, n=8, N=186; p(B-C)=0.0175, p(B-G)=0.0147) were detected in axonal growth cones in comparison to CNTF (0.62 ± 0.06, Q_2_ 0.63, n=5, N=148) and GDNF (0.63 ± 0.10, Q_2_ 0.72, n=6, N=211) (two independent experiments with n>5). (D) Representative images of growth cones of *trkBTK^+/+^* and *trkBTK^-/-^* motoneurons cultured on laminin-221/211 for 5 days *in vitro* and stained against Glu-α- (green, glutamylation) and tyrosinated- (magenta, Tyr, YL1/2) tubulin and F-actin (yellow) (scale bar: 5µm). There were no significant differences between both genotypes in Glu-Tub and Tyr-Tub staining, whereas F-actin levels appeared reduced. (E) Similar findings with respect to Glu- (green) and Tyr-Tub (magenta) were obtained with BDNF-, CNTF- or GDNF-treated motoneurons (scale bar: 5µm). (F) Control growth cones appeared to reveal higher levels of F-actin (magenta) in relation to tau (green) on laminin-221/211 than on laminin-111, whereas *trkTK^-/-^* growth cones exhibited comparable F-actin/tau intensities independently of the used laminin isoform (scale bar: 5µm). On laminin-221/211 a difference in F-actin/tau was indicated between control and *trkBTK^-/-^* growth cones, whereas on laminin-111 signals appeared comparable. (C, E) Modified from @Dombert, B. (2014). Molecular mechanisms underlying defective axonal growth and presynaptic differentiation in spinal muscular atrophy. Universitaet Wuerzburg.

**Figure S5: Specific effects of BDNF on axonal growth cones of motoneurons cultured on laminin-221/211 and laminin-111.** (A) Representative images of non-pulsed and BDNF-pulsed axonal growth cones of motoneurons cultured on laminin-221/211 for 5 days *in vitro* and stained against trkB (scale bar: 5µm). Upon BDNF pulse trkB immunoreactivity was moderately increased, particularly at growth cone protrusions. (B) Validation of obtained trkB signals by shRNA-mediated lentiviral knockdown (trkB – magenta, APP – blue, GFP – green, scale bar: 10µm). In shtrkB-transduced motoneurons trkB immunoreactivity was highly reduced in comparison to non-transduced and GFP-transduced cells (2 independent experiments). (C) BDNF-induced trk phosphorylation was also detectable in whole cell lysates produced from embryonic motoneurons activating AKT and MAPK signaling pathways which was accompanied by phosphorylation of LIM kinase 1 and 2 and cofilin at Ser 3. (D) Filamentous to globular actin separation revealed a shift toward filamentous β-actin in BDNF-pulsed motoneuron lysates in comparison to non-pulsed controls (two independent experiments with n=3). (E) On laminin-111 BDNF application resulted in a moderate increase of pTrk levels (magenta) in relation to SynPhys (green) in axonal growth cones of embryonic motoneurons in comparison to non-pulsed controls (two independent experiments: no pulse – n=3, N=56; 5’ BDNF – n=4, N=68; scale bar: 5µm). (F) Representative images of non-pulsed and BDNF-pulsed growth cones of embryonic motoneurons cultured for 5 days *in vitro* on laminin-111 and stained against β-actin (green), F-actin (magenta) and tau (blue) (scale bar: 5µm). Upon BDNF pulse β-actin/tau immunoreactivity appeared increased in comparison to non-pulsed controls, whereas F-actin/tau levels were comparable in both conditions (two independent experiments: no pulse – n=3, N=48; 5’ BDNF – n=4, N=68). (B) Modified from @Dombert, B. (2014). Molecular mechanisms underlying defective axonal growth and presynaptic differentiation in spinal muscular atrophy. Universitaet Wuerzburg.
